# First Plant Cell Atlas symposium report

**DOI:** 10.1002/pld3.406

**Published:** 2022-06-08

**Authors:** Selena L. Rice, Elena Lazarus, Christopher Anderton, Kenneth Birnbaum, Jennifer Brophy, Benjamin Cole, Diane Dickel, David Ehrhardt, Noah Fahlgren, Margaret Frank, Elizabeth Haswell, Shao‐shan Carol Huang, Samuel Leiboff, Marc Libault, Marisa S. Otegui, Nicholas Provart, R. Glen Uhrig, Seung Y. Rhee, The Plant Cell Atlas Consortium

**Affiliations:** ^1^ Department of Plant Biology Carnegie Institution for Science Stanford California USA; ^2^ Environmental Molecular Sciences Division Pacific Northwest National Laboratory Richland Washington USA; ^3^ Center for Genomics and Systems Biology New York University New York New York USA; ^4^ Department of Bioengineering Stanford University Stanford California USA; ^5^ Lawrence Berkeley National Laboratory Berkeley California USA; ^6^ Octant Emeryville California USA; ^7^ Donald Danforth Plant Science Center St. Louis Missouri USA; ^8^ Department of Plant Biology Cornell University Ithaca New York USA; ^9^ Department of Biology Washington University in St. Louis St. Louis Missouri USA; ^10^ Department of Botany and Plant Pathology Oregon State University Corvallis Oregon USA; ^11^ Department of Agronomy and Horticulture University of Nebraska‐Lincoln Lincoln Nebraska USA; ^12^ Department of Botany University of Wisconsin‐Madison Madison Wisconsin USA; ^13^ Department of Cell and Systems Biology/Centre for the Analysis of Genome Evolution and Function University of Toronto Toronto Ontario Canada; ^14^ Department of Science University of Alberta Edmonton Alberta Canada

**Keywords:** data science, live imaging, plant cell atlas, proteomics, single‐cell sequencing, spatial transcriptomics

## Abstract

The Plant Cell Atlas (PCA) community hosted a virtual symposium on December 9 and 10, 2021 on single cell and spatial omics technologies. The conference gathered almost 500 academic, industry, and government leaders to identify the needs and directions of the PCA community and to explore how establishing a data synthesis center would address these needs and accelerate progress. This report details the presentations and discussions focused on the possibility of a data synthesis center for a PCA and the expected impacts of such a center on advancing science and technology globally. Community discussions focused on topics such as data analysis tools and annotation standards; computational expertise and cyber‐infrastructure; modes of community organization and engagement; methods for ensuring a broad reach in the PCA community; recruitment, training, and nurturing of new talent; and the overall impact of the PCA initiative. These targeted discussions facilitated dialogue among the participants to gauge whether PCA might be a vehicle for formulating a data synthesis center. The conversations also explored how online tools can be leveraged to help broaden the reach of the PCA (i.e., online contests, virtual networking, and social media stakeholder engagement) and decrease costs of conducting research (e.g., virtual REU opportunities). Major recommendations for the future of the PCA included establishing standards, creating dashboards for easy and intuitive access to data, and engaging with a broad community of stakeholders. The discussions also identified the following as being essential to the PCA's success: identifying homologous cell‐type markers and their biocuration, publishing datasets and computational pipelines, utilizing online tools for communication (such as Slack), and user‐friendly data visualization and data sharing. In conclusion, the development of a data synthesis center will help the PCA community achieve these goals by providing a centralized repository for existing and new data, a platform for sharing tools, and new analytical approaches through collaborative, multidisciplinary efforts. A data synthesis center will help the PCA reach milestones, such as community‐supported data evaluation metrics, accelerating plant research necessary for human and environmental health.

## 1 INTRODUCTION

With the rapidly changing climate and growing population, plant science is becoming increasingly vital for agriculture, environmental stewardship, energy, healthcare, manufacturing, and technology. To aid in solving fundamental problems in plant biology, the Plant Cell Atlas (PCA) initiative aims to provide a conceptual and technological framework to identify and map all the components of plant cells from nano‐ to macro‐scale resolutions so that one day we may be able to build and design plant cells. Being able to engineer plant cells is just the beginning of building a sustainable, plant‐based future.

To work toward this ambitious future, the PCA is building a global community with diverse scientific, technical, and educational backgrounds to share passions and knowledge. In doing so, the PCA will be positioned at the frontier of plant science, leveraging innovative and emerging technologies to push the boundaries of science and technology. The multidisciplinary nature of the PCA has enormous potential for accelerating our knowledge of plant cells and how they connect to the whole organism and for translating fundamental discoveries to agriculturally important crops across the globe. The core PCA infrastructure should function as a data resource onto which services and tools are built to enable this transformation. Key elements of the PCA platform will be the collection, standardization, biocuration, integration, analysis, and visualization of data at single‐cell resolutions.

On December 9 and 10, 2021, the PCA held a virtual symposium to explore the challenges and opportunities of the PCA community (see Appendix S1 for the full program and Appendix S2 for summaries of the oral presentations). The symposium, which is summarized in this report, brought together nearly 500 researchers, policymakers, and other experts from around the world (see Appendix S5 for symposium participants) for 2 days of scientific talks and discussion on the possible development of a data synthesis center to address the needs of the PCA community (see Appendix S3 for individual responses ordered by upvoting). About 60% of the participants were early‐career researchers (i.e., graduate students, postdoctoral researchers, or assistant faculty), and about 40% were non‐US based. The attendees represented a total of 48 countries. The symposium was well received by the community, with 90% of respondents to our feedback survey reporting that they found the symposium helpful or very helpful to their research (see Appendix S4 for feedback data).

Ultimately, by promoting wider access to data and tools and enabling the synthesis of data, the PCA has the potential to empower researchers—even those with limited resources—to study meaningful research questions in plant biology with innovative solutions, connect with stakeholders beyond the scientific community, and address society's most pressing challenges in agriculture, food security, bioenergy, resource management, and environmental stewardship.

## 2 DATA ANALYSIS TOOLS AND ANNOTATION STANDARDS

The PCA's vision of developing a platform that brings together data at multiple scales, from molecular to cellular to organismal, comes with many challenges. While the vision of a PCA will require a large amount of high quality data from a variety of technologies, studies which generate this data are being performed with rapidly increasing frequency and with ever‐greater data output since the first plant single‐cell studies were published in 2018–2019 (Cole et al., 2021; Cuperus, 2021). As technology becomes increasingly accessible, we expect that the problem of the PCA will become less about data availability, and more about data analysis, integration, and interpretation. Despite the availability of a range of data types and current efforts to compile re‐analyzed single‐cell expression data (Papatheodorou et al., 2020), the lack of centrality in multi‐data deposition limits the accessibility and use of these datasets. Moreover, without data and metadata standardization and curation, both existing and new data cannot be integrated in a meaningful way.

Thus, to support the PCA community's research, data analysis tools and annotation standards are needed. Many considerations must be made for harmonizing and collectively interpreting divergent datasets, such as ensuring that all relevant metadata and annotations are included in published studies, establishing standard data exchange practices and file formats, and developing and updating various tools for comparing omics data between plant species to generate more “universal” annotation standards. Standardization of methods and data processing, such as sequence or proteomic data processing, is needed for quality control. More efforts should focus on establishing plant species annotations and implementing version controls for the annotations (Sumner et al., 2007; Van Bel et al., 2022), as well as keeping data repositories as up to date as possible.

A synthesis center‐scale investment could also create a repository, or integrate with existing resources such as Planteome (Cooper et al., 2018), that lists plants and their specific cell types and allows users to deposit, access, and compare data. To streamline the transfer of information between experimental systems, cell‐type marker homologs across species should be investigated. While the Plant Cell Marker DataBase already incorporates some of these functionalities (Jin et al., 2022), a platform enabling open‐access gene function curation would also benefit the plant biology community.

To accelerate the development of data analysis tools, a sandbox‐type environment would enable users to upload and use analysis tools as they become available. A gold‐standard, multimodal dataset would also advance data analysis method development and benchmarking. Finally, approaches for integrating single‐cell data in gene discovery platforms such as Knetminer (Hassani‐Pak et al., 2021) should be considered.

## 3 COMPUTATIONAL EXPERTISE AND A CYBER‐INFRASTRUCTURE

The PCA community will require resources, including computational expertise and cyber‐infrastructure, to advance the science and meet its goals for broader impacts. For instance, a centralized and extensible single‐cell visualization browser would ensure that each new study does not need to create its own browser. Data visualizations and dashboards should be easy to use for casual data browsing, and datasets and packages should be compatible with mainstream analysis packages and pipelines (e.g., BSGenome (Pagès, 2021) and Signac/Seurat (Hao et al., 2021; Stuart et al., 2021) for model/crop species). Reproducible single‐cell genomics pipelines, using tools such as Nextflow (Di Tommaso et al., 2017) and Docker (Merkel, 2014), could allow documentation as a notebook with containerized software images.

Methods that integrate different single‐cell data modalities, for example, single‐cell RNA sequencing and spatial transcriptomics, are needed to assemble 3D/4D models of plant organs. In conjunction with computational resources, a repository should be developed to share experimental protocols (e.g., nuclei isolation and tissue preparation) specialized for plant species and tissue types. This repository of protocols, along with a community of experts for advice, would be immensely useful in increasing the accessibility of technologies. The online infrastructure should include a list of reporter lines that are valuable for cell‐type/cluster annotation of single‐cell RNA sequencing data and gold‐standard datasets for teaching and developing new methods. Finally, the database should incorporate cell‐type marker genes and their corresponding orthologs, expressologs, and matches across species, synteny maps of good markers, and a repository to record findings if marker genes translate across species. While gene orthology viewers, are part of the several platforms (e.g., PLAZA, EnsemblPlants, Phytozome, and Gramene) and are already providing an ensemble approach to identify gene orthology relationships (Goodstein et al., 2012; Howe et al., 2020; Tello‐Ruiz et al., 2021; Van Bel et al., 2022), orthology tools also integrating single‐cell transcriptome information across species are not yet available. Furthermore, orthology‐based projection of gene annotation approach has limitations in providing accurate functional annotation for gene family members (Naithani et al., 2021; Tang et al., 2019) and manual biocuration of plant gene families will help analysis of PCA datasets as well.

Collaborative efforts will help leverage the potential of individual labs in dissecting cellular complexity. To facilitate collaborations, the PCA website should provide a list of participating labs and community members, along with their specific expertise. An online space should also be provided to enable discussion and troubleshooting among the plant single‐cell sequencing community.

Finally, training scientists in the analysis of single‐cell plant datasets should be emphasized, potentially in the form of summer schools or workshops. For instance, hands‐on bioinformatics workshops could center on the “hows and whys” of certain analyses. As not everyone can attend a physical workshop, the PCA should consider ways of distributing training materials that are free and accessible to all, or holding hybrid or virtual workshops. Outreach efforts could also focus on incorporating plant cell genomics into undergraduate and K–12 education. Moreover, an increased social media presence will help get the public excited about plant cells.

## 4 NOVEL MODES OF COMMUNITY ORGANIZATION AND ENGAGEMENT

The PCA community must explore novel modes of organization and engagement to catalyze new ideas, research directions, and discoveries in a time of rapid change. Monthly or bi‐monthly meetings on specific topics or journal clubs would facilitate discussions and collaborations. Existing PCA Slack channels could be leveraged for these communications. “Brainstorming” days, that is, pre‐defined days of synchronous discussion, could concentrate on specific topics, questions, or ideas on a platform like Slack or Discourse. Finally, events focused on early‐career researchers would help build a community among graduate students, postbacs, and postdoctoral fellows.

The PCA could consider ideation platforms such as Hype or Yambla to establish an idea caretaker community and processing workflow to enable ideas to be developed into projects with expert guidance. These tools could serve as ideation‐ or solution‐providing platforms to spark creativity globally and to develop ideas.

In terms of sharing data, the Single Cell Portal might provide a template for sharing data and analysis pipelines. Another potential model is DataCite, which takes an interactive approach to making their roadmap development public.

Finally, it would be valuable to further connect with other atlases to model their data streams. For instance, the Human Cell Atlas has subgroups with specific goals and challenges; this approach could help to further organize the PCA community. Identifying and organizing subgroups interested in the same types of data collection or analysis around common goals could encourage collaboration.

## 5 ENGAGING A BROAD COMMUNITY

The PCA community must ensure that individuals and groups who are not regular participants due to disciplinary barriers, cultural differences, or resource limitations are included. To this end, efforts should focus on open sharing of metadata, data, and code. Beyond data and resource sharing, virtual opportunities can help broaden the reach of the PCA. Events could enable natural networking and discussion through virtual interfaces that emulate real conference experiences of “walking into someone.” Because research can be expensive, the PCA could consider developing a virtual Research Experience for Undergraduates program for single‐cell biology (e.g., https://nsurp.org/). In addition to specialized virtual experiences, introductory online workshops and webinars could cater to high school and college students with no research experience. The PCA could also focus on finding ways to reduce the cost of conducting research, either by developing inexpensive, accessible technologies or by lobbying for grants to fund projects. Such efforts should prioritize proposals with economic or other tangible benefits, such as single‐cell omics on traits (e.g., disease and stress tolerance) of common staple crops or commercially grown plant varieties. This approach would better engage breeders and farmers in the PCA.

To reach underserved organizations and countries, the PCA could create a panel of experts to serve as advisors. Special planning groups could meet to understand the needs of communities of interest and consider forming local PCA chapters. The PCA could develop introductory bioinformatics workshops for underserved high school or college students.

Finally, it will be necessary for the PCA community to promote an inclusive and supportive culture that fosters conversations and encourages everyone to contribute. Discussions should center on “imposter syndrome” and other emotional challenges that are common in research settings. There should also be a clear code of conduct with zero tolerance of harassment.

## 6 RECRUITING, TRAINING, AND NURTURING NEW TALENT

It is essential to develop and support the next generation of plant scientists to help achieve the goals of the PCA; this will seed paradigm‐shifting discoveries in the future. New students and talent can be brought in from other disciplines, helping to diversify the thought base. Platforms, collaborations, and events could recruit and engage such researchers. To foster cross‐disciplinary connections, engineering, bioinformatics, computational science, and information technology courses can include plant science and, vice versa, plant biology programs can include these disciplines. The PCA could also develop flyers on exciting plant science technologies to distribute to engineering, physics, computation, and chemistry departments. Research focused on human biology, evolution, or ecology could leverage the ease of experimentation in plant biology, again promoting cross‐disciplinary collaborations. These collaborations could arise through the sharing of tools and resources from the PCA.

Another way to attract and nurture new talent is to develop methods for familiarizing a broader range of people with single‐cell datasets. The PCA could hold specialized workshops, write periodic review articles on single‐cell technologies, and establish opportunities for training. Internships for graduate students could be promoted through commercial providers such as 10X Genomics or Resolve Biosciences. In addition, the PCA could promote the availability of programs at user facilities such as the Environmental Molecular Sciences Laboratory to learn about new technologies. The PCA could inform public and private funding institutions about the importance of specialized technologies for plant tissues and lobby for more funding to this area of plant biology to draw more researchers to the field. Finally, the PCA could inspire the development of exciting new technologies and ideas through competitions, such as a hackathon contest, open challenges on Kaggle or other data repositories, or a plant science competition in partnership with philanthropy and industry.

It will also be critical to get people excited about plant science during early education. Efforts should focus on exposing K–12 students to gardening and emerging technologies for growing plants, such as vertical farming, hydroponics, and shipping container farming. An exciting presence on social media can also help spark curiosity and interest in non‐scientists and younger students who have not yet chosen a discipline. The PCA should consider engaging with scientific communication students (i.e., journalism or media departments) to develop inspiring content for the public. The PCA could also find ways to include plant cell science in core undergraduate education through influencing major textbook authors, developing conceptual and hands‐on educational materials, and assembling undergraduate career path materials to increase awareness of jobs in plant sciences.

## 7 SERVING THE WIDER SCIENTIFIC COMMUNITY

A necessary goal of the PCA must be to inform the wider scientific community on the value of using single‐cell approaches to better understand plant biology and apply this knowledge to design new strategies to improve crop performance. To help achieve this goal, there is a need to connect the single‐cell research community with the traditional plant biology community. Moreover, this mindset can be developed through education from high schools to graduate schools.

Beyond the plant biology field, the PCA can apply its data, resources, and tools to help clarify biological mechanisms in other organisms. Attention should focus on shaping the view of plants as essential tools for understanding cell biology in general. By building a community and focus, the PCA can help garner the attention of animal and microbial biologists as well as applied science and industry researchers. Opportunities to make these connections include international conferences that bring together leading minds across biological systems, such as Advances in Genome Biology and Technology conferences or Gordon Research Conferences.

Finally, outreach should occur outside of the scientific community to reach important stakeholders such as breeders and growers and seek out stakeholders in regions not currently well represented in the PCA, such as the South and Central United States. Many universities and states have outreach and extension offices that can connect researchers directly with growers. The PCA should leverage these opportunities in order to engage a broader community.

## 8 CONCLUSIONS

The PCA will bridge gaps in knowledge, providing critical location, dynamics, and interaction information about molecules at the cellular and subcellular levels, to enable quantitative understanding and engineering of plant cells. To achieve these goals, a data synthesis center could assist in synthesizing the wealth of existing data, resources, and tools by harmonizing and collectively interpreting divergent datasets, developing new analytical approaches and tools, building models and theories, and integrating knowledge from within and across various disciplines. A data synthesis center could help the PCA reach milestones such as (1) developing community‐supported evaluation metrics; (2) establishing data benchmarking platforms and data analysis challenges; and (3) performing data simulation, validation, and curation. Reaching these milestones will greatly accelerate plant research and place us in a better position to tackle the societal challenges posed by a growing human population and climate change.

## CONFLICT OF INTEREST

The Authors did not report any conflict of interest.

## Supporting information


**Figure S1: How relevant and helpful was the 2021 PCA Symposium for your research?** Results from survey question #1 (N = 61 responses). The question was posed with a 5‐point likert scale ranging from 1 (Not Helpful) to 5 (Very Helpful). 55 respondents reported a rank of 4 or 5 (~90%).
**Figure S2: Which areas of your work was this symposium helpful for, if any?** Results from survey question #2 (N = 57 responses, where 27 respondents chose more than one area).
**Figure S3: How satisfied were you with the following workshop components?** Results from survey question #3 (N = 61 responses). The question was posed with a 5‐point likert scale ranging from 1 (Very Dissatisfied) to 5 (Very Satisfied).
**Table S1. Any overall feedback for the event (what worked well and what didn't work as well)?** Results from survey question 4 (N = 32 responses). We coded the responses into 3 categories: General Positive Response, Constructive Criticism, and General Criticism. We then identified 6 subcategories of Constructive Criticism comments: poster sessions (3), community discussions (4), networking opportunities (2), talk titles in accelevents (4), time zone (2), and other (4). These comments are intentionally unedited from the survey to retain the original input from the community.
**Table S2. What are the types of technologies you would like to hear about in a future PCA webinar?** Results from survey question 5 (N = 24 responses). We coded the responses into 4 categories: single cell omics (10), data analysis (4), imaging (5), and other (5). These comments are intentionally unedited from the survey to retain the original input from the community.
