# Supplementary material for: First Plant Cell Atlas symposium report
Source: Plant Direct. 2022 Jun 8;6(6):e406. doi: 10.1002/pld3.406 (PMC9219010; doi:10.1002/pld3.406)
Supplement: Supplementary file 1 — Figure S1: How relevant and helpful was the 2021 PCA Symposium for your research? Results from survey question #1 (N = 61 responses). The question was posed with a 5‐point likert scale ranging from 1 (Not Helpful) to 5 (Very Helpful). 55 respondents reported a rank of 4 or 5 (~90%). Figure S2: Which areas of your work was this symposium helpful for, if any? Results from survey question #2 (N = 57 responses, where 27 respondents chose more than one area). Figure S3: How satisfied were you with the following workshop components? Results from survey question #3 (N = 61 responses). The question was posed with a 5‐point likert scale ranging from 1 (Very Dissatisfied) to 5 (Very Satisfied). Table S1. Any overall feedback for the event (what worked well and what didn't work as well)? Results from survey question 4 (N = 32 responses). We coded the responses into 3 categories: General Positive Response, Constructive Criticism, and General Criticism. We then identified 6 subcategories of Constructive Criticism comments: poster sessions (3), community discussions (4), networking opportunities (2), talk titles in accelevents (4), time zone (2), and other (4). These comments are intentionally unedited from the survey to retain the original input from the community. Table S2. What are the types of technologies you would like to hear about in a future PCA webinar? Results from survey question 5 (N = 24 responses). We coded the responses into 4 categories: single cell omics (10), data analysis (4), imaging (5), and other (5). These comments are intentionally unedited from the survey to retain the original input from the community. [file PLD3-6-e406-s001.pdf]

Appendix S1: Symposium Schedule

December 9, 2021

Oral Session 1

7:00 AM - 7:20 AM	Yvon Jaillais (ENS Lyon, France)<br>Talk Title: *Anionic phospholipids across scales: from plasma membrane nanodomains to plant development*
7:30 AM - 7:40 AM	Hilde Nelissen (VIB-Ghent University, Belgium)<br>Talk Title: *An* in situ *sequencing approach maps PLASTOCHRON1 at the boundary between indeterminate and determinate cells*
7:45 AM - 7:55 AM	Gergo Palfalvi (National Institute for Basic Biology, Japan)<br>Talk Title: *Evolution and development of carnivorous plant leaves*
8:00 AM - 8:30 AM	**Community Discussion 1**<br>What data analysis tools and annotation standards are needed to support and centralize the community's research?

Poster Session 1

8:30 AM - 9:30 AM Poster #s: 01-08

Oral Session 2

9:40 AM - 9:50 AM	Xiaosa Xu (Cold Spring Harbor Laboratory, USA)<br>Talk Title: *A high-resolution single-cell atlas of shoot meristems at cross-species and multimodal levels*
10:00 AM - 10:20 AM	Stefania Giacomello (SciLifeLab - KTH, Sweden)<br>Talk Title: *Unveiling spatial host–microbiome interactions by applying spatial metatranscriptomics*
10:25 AM - 10:35 AM	Lachezar Nikolov (University of California, Los Angeles, USA)<br>Talk Title: Tabula floris – *a panoramic view of the cell types of the flower*
10:40 AM - 11:10 AM	**Community Discussion 2**<br>What types of resources, including computational expertise and cyber-infrastructure, will the community need to best advance the science and meet its goals for broader impacts?

Poster Session 2

11:50 AM - 12:30 PM Poster #s: 09-13

Oral Session 3

12:30 PM - 12:50 PM	Mathew G. Lewsey (La Trobe University, Australia)<br>Talk Title: *Regulatory dynamics of germinating seeds from bulk tissue to single-cell resolution*
1:00 PM - 1:10 PM	Marc Libault (University of Nebraska-Lincoln, USA)<br>Talk Title: *Single-nucleus RNA and ATAC sequencing reveals the impact of chromatin accessibility on gene expression in Arabidopsis roots at the single-cell level*
1:15 PM - 1:25 PM	Bruno Guillotin (New York University, USA)<br>Talk Title: *Mapping cellular divergence in crop plants via comparative single-cell and nuclei analysis*
1:30 PM - 2:00 PM	**Community Discussion 3**<br>What novel modes of organization and engagement will the community explore to catalyze new ideas, research directions, and discoveries in a time of rapid change?

December 10, 2021

Oral Session 4

7:00 AM - 7:20 AM	Nancy George (European Molecular Biology Lab-EBI, UK)<br>Talk Title: *Single-cell expression atlas: Archiving and visualization of plant single-cell data*
7:30 AM - 7:40 AM	Vimal Kumar Balasubramanian (Pacific Northwest National Laboratory, USA)<br>Talk Title: *Integration of Cell Type-Specific Omics Analysis Towards a Spatiotemporal Understanding of Molecular Responses to Abiotic Stresses in Poplar*
7:45 AM - 7:55 AM	Camilla Ferrari (VIB-Ghent University, Belgium)<br>Talk Title: *Identification of cell-type specific gene regulatory networks in plants using MINI-EX, a Motif-Informed Network Inference method based on single-cell EXpression data*
8:00 AM - 8:30 AM	**Community Discussion 4**<br>How will the community ensure that individuals and groups who are not regular participants due to disciplinary barriers, cultural differences, or resource limitations are included in the PCA?

Poster Session 3

8:30 AM - 9:30 AM	Poster #s: 14-20

Oral Session 5

9:40 AM - 10:00 AM	Kevin Cox, Jr. (Donald Danforth Plant Science Center, USA)<br>Talk Title: *Integrating spatial transcriptomics and 3D imaging in plants*

10:10 AM - 10:20 AM	Margot Bezrutczyk (Lawrence Berkeley National Lab, USA)<br>Talk Title: *Mapping plant-mycorrhizal interactions with spatial transcriptomics and single-nuclei sequencing*
10:25 AM - 10:35 AM	Maite Colinas (Max Planck Institute for Chemical Ecology, Germany)<br>Talk Title: *Plant response to "friend or foe": a cell-type-specific proteomics approach*
10:40 AM - 11:10 AM	**Community Discussion 5**<br>In what ways will the community recruit, train, and nurture new talent to seed paradigm-shifting discoveries in the future?

Poster Session 4

11:50 AM - 12:30 PM   Poster #s: 21-25

Oral Session 6

12:30 PM - 12:50 PM	Federica Brandizzi (Michigan State University, USA)<br>Talk Title: *The plant endoplasmic reticulum at the interface with other organelles: How and what for?*
1:00 PM - 1:10 PM	Constance Le Gloanec (University of Montreal, Canada)<br>Talk Title: *Cell-type-specific behaviors contribute to leaf growth variability in* Arabidopsis
1:15 PM - 1:25 PM	Michael Taylor (Pacific Northwest National Laboratory, USA)<br>Talk Title: *Development of targeted single cell analysis and metabolic imaging of plants using high spatial resolution laser ablation electrospray ionization mass spectrometry*
1:30 PM - 2:00 PM	**Community Discussion 6**<br>How will the outcomes of the community's efforts serve the wider scientific community?

Appendix S2: Summaries of the Presentations

DAY 1 - Dec 9, 2021
Oral Session 1

1. **Anionic phospholipids across scales: from plasma membrane nanodomains to plant development**
   Speaker: **Yvon Jaillais**, ENS Lyon, France

Dr. Jaillais's lab investigates anionic phospholipids, which act as strong signals within the membrane and can recruit proteins in different membranes of the cell. Ultimately, the goal of their work is to understand anionic lipids across scales (i.e., the nanoscale, subcellular and cellular scale, and tissue and organ scale).

In particular, Dr. Jaillais has studied phosphatidylserine (PS). His group used an intracellular PS biosensor to determine the localization of PS and found that it accumulates at the cellular membrane. Analysis of the root indicated that PS is not present at the same concentration in different cells. In the elongation and differentiation zones of the root, the PS concentration is lower at the membrane than in the division zone. What function does this PS gradient perform? Using a genetic approach to mutate the PS synthase I (PSSI) enzyme that generates PS, Dr. Jaillais's group found a strong developmental phenotype in plants lacking PS. In addition, Dr. Jaillais used artificial microRNA to lower PS levels as well as PSSI overexpression to increase PS levels. These variations in PS level revealed that PS grades the root gravitropic response.

The Rho-like GTPase 6 (ROP6) molecule is known to trigger the gravitropic response by regulating trafficking and microtubule dynamics as well as auxin transport and growth. Thus, Dr. Jaillais explored whether PS is involved in ROP6-mediated functions. Indeed, tuning the PS level graded the ROP6 signaling output. However, loss of PS had little effect on ROP6 plasma membrane targeting, complicating the interpretation of how PS controls ROP6 function. Instead, experiments using azimuthal total internal reflection fluorescence microscopy, photoactivated localization microscopy (PALM), and single-particle tracking PALM revealed that PS is involved in the distribution of ROP6 within the plasma membrane. These approaches made it possible to study the single-molecule dynamics of ROP6 in the plasma membrane of live plants. Specifically, these efforts revealed that PS regulates auxin-mediated ROP6 nanoclustering.

Key takeaways: PS variations tune ROP6 signaling and nanoclustering. Moreover, PS levels at the plasma membrane vary during cell differentiation, which may provide a gradient for regulating ROP6.

2. **An *in situ* sequencing approach maps PLASTOCHRON1 at the boundary between indeterminate and determinate cells**

Speaker: **Hilde Nelissen**, VIB-Ghent University, Belgium

Plant growth is a continuous process that involves coordinated growth of different organs. Dr. Nelissen's group uses the maize seedling leaf as a model for organ growth because it exhibits both a spatial organization and temporal regulation of growth. Over several years, efforts in the lab have identified molecular players involved in this growth, including plastochron1 (PLA1). Targeted expression of this enzyme improves biomass and seed yield. Moreover, PLA1 is specifically expressed in the shoot apical meristem. *In situ* sequencing revealed spatial transcriptomics in the maize shoot apex and identified PLA1 at the border between undifferentiated and differentiated cells. These findings are consistent with the hypothesis that PLA1 plays a role in the timing of cell exit from an undifferentiated state.

In addition, molecular cartography enables the capture of molecular interactions at a higher resolution than *in situ* sequencing technology. Combining this approach with cell segmentation allowed PLA1 mapping to specific cells and defined co-expression with other molecules such as ANGUSTIFOLIA3. With an optimized spatial transcriptomics pipeline for sectioning, imaging, segmentation, and data analysis, this method has many potential future applications.

Key takeaways: Spatial transcriptomics is an emerging field, is highly complementary to single-cell RNA sequencing, and can be a valuable tool in developmental biology. Different types of analyses are possible, depending on the platform, for a larger or smaller region of interest.

3. **Evolution and development of carnivorous plant leaves**
   Speaker: **Gergo Palfalvi**, National Institute for Basic Biology, Japan

Although carnivorous plants cross multiple evolutionary clades, they share physiological and morphological adaptations. The heterophyllous pitcher plant *Cephalotus follicularis* provides an ideal system to study these adaptations, as it can grow both carnivorous pitcher leaves and photosynthetic flat leaves. Environmental factors (e.g., nutrition and temperature) can be applied to modulate which leaf types are formed. In early primordial stages, the two leaf types are indistinguishable. However, in the P2–3 stages, morphological features begin to emerge in the pitcher leaves.

Carnivory is related not only to morphology but also to function, which is highly related to specialized cell types. For example, small glands are similar to guard cells but are immobile and have a strong digestive ability, large glands are multicellular and have suberinized cell walls, and imbricate hairs prevent insects from escaping. Thus, understanding the evolution of these cell types is key to clarifying the evolution of carnivory in these plants. To elucidate how these cell types emerged in evolution and development, Dr. Palfalvi and colleagues aimed to develop a single-cell map of *Cephalotus follicularis.*

Single cell transcriptomics of developing and mature leaf types could recover 90,000 cells. Known markers could be applied to identify many of these cell types. In a gland-like cell cluster, some subclusters are specific to the pitcher leaves and express known digestive enzymes; thus, these are likely the cell types responsible for digestion. These data also led to new marker genes associated with digestive cells. Many pitcher-specific cell types were related to epidermal cell types, some of which appear to represent the imbricate hairs. During the development of this cell type, early cell clusters emerged in the pitcher leaves, which are involved in stress response and possibly growth inhibition. Later developmental cell clusters were related to modified cell wall properties. Thus, the cell types around the entrance and inside the pitcher should be restricted in growth, and stress-response-related genes might regulate this process, providing insights into the development of the pitcher shape.

Key takeaways: Single-cell sequencing and advanced imaging techniques can enhance our understanding of non-model organisms and the evolution of novel cell types in carnivorous plants.

Oral Session 2

1. **A high-resolution single-cell atlas of shoot meristems at cross-species and multimodal levels**
   Speaker: **Xiaosa Xu**, Cold Spring Harbor Laboratory, USA

The plant shoot meristem determines the architecture of a plant; thus, understanding this system is important to inform future work seeking to design crops with improved performance. Dr. Xu's work aims to clarify how the shoot meristem is regulated in maize and Arabidopsis throughout its lifecycle. Localized expression of master regulators drives plant development. Dr. Xu first performed single-cell RNA sequencing (RNA-seq) on developing maize ear and identified and validated 12 cell groups marking distinct cell-types or developmental domains. However, the rare stem cells were not recovered. A similar challenge was also reported in an Arabidopsis shoot meristem single-cell study. Thus, Dr. Xu finely dissected the developing maize ear tip to enrich stem cells. Dr. Xu also profiled Arabidopsis *apetala1; cauliflower* double mutants, which proliferate to produce many shoot meristems. Dr. Xu successfully recovered stem cells for both species and identified and validated conserved stem cell markers. To further understand stem cell proliferation in maize, Dr. Xu then profiled the ear tip of *fea3; Zmcle7* double mutants, which were strongly fasciated, and identified a family of metabolic genes that play a role in shoot stem cell development.

Dr. Xu also examined plant gene regulation at single-cell level. Accessible chromatin regions are often associated with regulatory elements that control gene expression. Single-cell ATAC sequencing (ATAC-seq) can probe this level of regulation. By integrating maize single-cell RNA-seq and single-cell ATAC-seq datasets of developing

maize ear and tassel, Dr. Xu identified cell-type-specific accessible chromatin regions associated with gene expression in maize inflorescences. Future work using CRISPR to generate *cis*-regulatory alleles will aid in elucidating the function of these regions and ultimately improving crop performance.

Key takeaways: Single-cell RNA sequencing is highly effective for capturing plant stem cell niches. Integrating single-cell RNA sequencing and ATAC sequencing provides essential information about accessible chromatin regions associated with gene expression in plants.

2. **Unveiling spatial host–microbiome interactions by applying spatial metatranscriptomics**
   Speaker: **Stefania Giacomello**, SciLifeLab - KTH, Sweden

Dr. Giacomello's research focuses on how cell gene expression influences the spatial organization of cells and how the location of cells influences cell communication and interactions. Originally developed for animal tissues, spatial transcriptomics has been applied to plants to study high-resolution, spatially resolved profiles. 10X Genomics has commercialized this technology with a higher spatial resolution compared with the original development.

Recent work has focused on advancing this technology in order to study the spatial organization of microbes on *Arabidopsis* leaves. The goals were to clarify the host response to microbe clusters and to understand microbe–microbe abundances at the spatial level. To this end, Dr. Giacomello's group developed spatial metatranscriptomics (SmT). This technology provides three readouts, one each for the host, microbes, and fungi. Experiments using infiltrating mCherry-tagged *Pseudomonas* demonstrated that the SmT approach can capture spatial information of the microbe. To validate this approach, the group compared their results with those obtained via the gold standard of the field, amplicon sequencing. This comparison showed that the SmT array can capture more microbial information than amplicon sequencing. Quantified bacterial and fungal profiles showed consistency across leaves of the same plants.

Next, Dr. Giacomello's group investigated the spatial organization of microbes on outdoor leaves. Hotspot analysis revealed significant colocalization of microbes on tissue sections. The bacteria showed substantially more hotspots than fungi, but there were instances of colocalization between the bacteria and fungi. Overall, there was a consistent number of microbial interactions across leaves of the same plant. By associating microbial interactions with hotspot analysis, the group concluded that spatial locations drive intra- and inter-kingdom interactions. Finally, photosynthesis-related genes and immune-related genes exhibited high expression at microbial hotspot locations.

Key takeaway: The SmT approach can simultaneously provide host, bacterial, and fungal spatial information to visualize single or overall bacterial and fungal taxonomical datasets and to study microbe hotspots in relation to host responses.

3. ***Tabula floris* – a panoramic view of the cell types of the flower**
   Speaker: **Lachezar Nikolov, University of California, Los Angeles, USA**

Dr. Nikolov's lab studies the genetic basis of floral diversity. The flower is a highly compacted, determinate branch consisting of concentric whorls of organs with distinct identities. Floral organ identity manifests in specialized cell types, and although the organ identity program has been elucidated, we understand very little about these processes at the cellular level. Thus, Dr. Nikolov's lab has developed a single-cell atlas of the flower, termed the *Tabula Floris*. This library contains information for approximately 30,000 cells and 27,000 genes and reveals similarities and divergences among plant cell types. Detecting the expression of thousands of genes provides the opportunity to study gene co-expression networks. Dr. Nikolov's group has inferred gene networks for all floral gene populations, which feature both small and large networks. Importantly, the gene co-expression network for the entire flower revealed global hub genes that represent prominent regulators in the flower.

At the base of the floral organs, the floral nectaries consist of specialized secretory tissue. To study the nectary population, Dr. Nikolov's lab generated single-cell transcriptome profiles of the flowers of *crabs claw* mutants that lack nectaries and then pinpointed a specific cell cluster that was absent in these mutants. This cell population appears to be very metabolically active based on gene co-expression network analysis. This effort revealed that *SWEET9* and *TPS24* expression depends on *CRABS CLAW (CRC)*. Similarly, *CWINV4* expression depends on *CRC* in the nectary, however not in the companion cells. This finding suggests that factors other than *CRC* control expression in the companion cells. Indeed, the networks are non-overlapping between the companion cells and the nectary. Thus, single-cell perturbation approaches are powerful for studying gene regulation in different cells.

Key takeaway: A comprehensive catalog of floral cell types has led to crucial insights into the transcriptomes of rare cell types, diversity in known cell types, and new cell types. Floral gene co-expression network analyses of secretory cells can reveal master regulators and downstream targets.

Oral Session 3

1. **Regulatory dynamics of germinating seeds from bulk tissue to single-cell resolution**
   Speaker: **Mathew G. Lewsey,** La Trobe University, Australia

The Lewsey lab seeks to clarify how seed germination occurs and is regulated. It is critical that this process be correctly controlled and in tune with environmental conditions because if the seed germinates at the wrong time, the plant will not grow.

How much does gene expression change over time during germination and how is this process controlled? To answer this question, Dr. Lewsey's group performed bulk gene expression analysis and identified 24,283 differentially regulated genes during the germination process in Arabidopsis seeds. These genes fell into interesting clusters of regulation: a transient upregulated cluster during the transition from stratification in darkness to light, a late upregulated cluster during the metabolic transition of light, and a downregulated cluster during early germination. In concert with these processes, a broad loss of methylation occurs in the genome, and large dynamic changes correlate with the transitions in small RNAs. Although these data are useful for building transcriptional models, there are limitations to using a bulk approach. The Arabidopsis seed has multiple tissues and organs, which are all composed of different cell types. Moreover, these cell types perform different functions at different times. Thus, Dr. Lewsey's work has shifted toward single-cell approaches.

Recent work has focused on measuring gene expression in single Arabidopsis cells at three time points of germination (early, mid, and late). A comparison of bulk RNA sequencing between protoplast and the non-protoplast samples clarified the effect of protoplast isolation on Arabidopsis seeds. This effort identified several genes that were differentially regulated due to protoplast isolation but were not dependent on the germination time point. Next, a single-cell RNA sequencing experiment investigated different time points in order to identify clusters of similar cells and annotate cell identities. This step is challenging because there are no defined maps to serve as the ground truth for single-cell RNA sequencing in seeds. Instead, Dr. Lewsey's group combed through the literature to determine marker transcripts that identify cell types and annotated clusters on the resulting t-distributed stochastic neighbor embedding plots. The clusters were specific to provasculartture, cotyledon, hypocotyl, and radicle cells. The group then used these clusters to define new marker genes and validate the cluster annotations in plants. These experiments also led to the discovery of cluster transitions over time, likely representing transitions to different transcriptional cell states. For such a transition to occur, one would expect high levels of transcriptional activity to allow the cells to reprogram. Indeed, using RNA velocity as a metric to measure transcription, the group detected a high level of active transcription across the clusters, associated with cell state changes. Future work will focus on understanding how this movement along a transcriptional trajectory is controlled.

Key takeaways: Substantial remodeling of gene expression occurs during germination. As seeds are complex structures composed of many cell types, single-cell methods allow researchers to better characterize which cell types are active at different locations and time points during germination.

2. **Single-nucleus RNA and ATAC sequencing reveals the impact of chromatin accessibility on gene expression in Arabidopsis roots at the single-cell level**
   Speaker: **Marc Libault,** University of Nebraska-Lincoln, USA

Dr. Libault's lab uses isolated plant nuclei to characterize the transcriptome and patterns of chromatin accessibility in Arabidopsis cells. Nuclei present many advantages over protoplasts for characterizing plants at the single-cell level: they are easier to generate than protoplasts, they strongly minimize size discrimination in microfluidic platforms, and their RNAs better represent newly synthesized transcripts to capture initial cellular responses. Single-nucleus RNA sequencing technology enables researchers to characterize plant cell transcriptomes for identifying differentially expressed genes. With this approach, Dr. Libault's group revealed the transcriptome of new Arabidopsis root cell types and then performed functional annotation using root cell-type marker genes.

Dr. Libault's group also aims to elucidate how plant genomes are read, regulated, organized, and expressed, especially in response to environmental stresses, using single-nucleus omics technologies. Ultimately, this knowledge could be translated to improve crop productivity and quality. With this approach, Dr. Libault has interrogated the sequence of transcriptional events controlling plant development, plant cell differentiation, and plant cell responses to stress, allowing the development of accurate gene networks. For example, single-nucleus ATAC sequencing technology can reveal discrete changes in chromatin accessibility. Comparative analyses suggest a correlation between gene expression and chromatin accessibility. Therefore, transcriptomic and epigenomic changes occurring at the single-cell level make it possible to infer causal relationships with high accuracy.

The lab is expanding this work to include other species beyond Arabidopsis such as soybean, Medicago, and sorghum. Thus, single-nucleus omics technologies are applicable to different plant species and tissues for enhancing the functional characterization of plant genes.

Key takeaways: Isolated plant nuclei can provide biologically meaningful transcriptomic information at the single-cell level. Chromatin accessibility at the location of the transcription start site of selected genes can act as a molecular marker of cell-type identity.

3. **Mapping cellular divergence in crop plants via comparative single-cell and nuclear analysis**
   Speaker: **Bruno Guillotin**, New York University, USA

Within Dr. Birnbaum's lab, Dr. Guillotin has focused on comparing cell types across species to obtain access to a new evolutionary concept of cellular divergence. This work

started with a complete study of cell-type evolution across species. *Zea mays* is a well-studied plant with numerous marker genes and genetic tools available; thus, this work aimed to use this knowledge to study other interesting but less-studied plants, including *Sorghum bicolor* and *Setaria viridis*. Is it possible to map cell types from one species to another using the rich knowledge of one plant species (in this case, *Zea mays*)? Can we ultimately compare cell types across species to determine whether certain cell types are evolving faster than others? To begin addressing these questions, Dr. Guillotin and his colleagues utilized both single-cell and single-nucleus RNAseq approaches. Combining these approaches in all three plant species enabled the team to identify every cell type in the species. The group is now validating these cell types with a newly developed whole-mount *in situ* hybridization approach.

In addition, by comparing the cellular transcriptomes across species, Dr. Guillotin has analyzed differential evolution. In the three species analyzed, the epidermis layers showed stable transcriptomes. By contrast, the cortical layers showed lower conservation scores, indicating divergence across species. Interestingly, the columella was the most divergent of all the cell types and appears to be a quickly evolving cell type in *Zea mays* compared with *Setaria viridis*. Some of the most divergent genes were expressed in maize columella, with many of these genes involved in mucilage production. These findings are consistent with differences in these species in the routing of mucilage production.

Key takeaways: Performing direct cell-type identification across species enabled the detection of a rapidly evolving cell type and associated molecular processes. This approach is valuable for mapping traits in crop species.

DAY 2 - December 10, 2021
Oral Session 4

1. Single-cell expression atlas: Archiving and visualization of plant single-cell data

Speaker: **Nancy George**, European Molecular Biology Lab-EBI (EMBL-EBI), UK

The Gene Expression Team of EMBL-EBI, led by Irene Papatheodorou, is developing an array of functional genomics resources. The EBI collects data from the community and then validates and stores the data in an archive. The EBI also integrates and visualizes data for the research community. Annotare is a data submission tool that captures plant single-cell sequencing data, including technical, protocol, and annotation information. Submission templates are tailored to different experiments and technologies.

Once data are submitted, the data are available for analysis and visualization in the Single-Cell Expression Atlas. The goal of this freely available resource is to provide information on where and under what conditions different genes are expressed at a

single-cell level. The atlas includes analysis results for almost 6 million cells across 229 single-cell studies in 18 species, including human, mouse, and fly, and 19 plant datasets across 4 species. With this resource, users can explore questions such as where a particular gene is expressed at the single-cell level and whether a gene defines a specific cell population. For each dataset, the atlas also includes the underlying metadata, raw result files, associated publications, and analysis methods and workflows. The Single-Cell Expression Atlas also works in partnership with other atlases, such as the Human Cell Atlas.

Key takeaway: Resources such as the Single-Cell Expression Atlas are only possible because of species-specific atlas efforts such as the PCA that bring communities together and define minimum standards to allow the reanalysis of data generated by experts.

2. **Integration of cell-type-specific omics analysis towards a spatiotemporal understanding of molecular responses to abiotic stresses in poplar**
   Speaker: **Vimal Kumar Balasubramanian**, Pacific Northwest National Laboratory, USA

Abiotic stresses, such as heat, drought, and salinity, are major challenges in future food and bioenergy crop production. Within Dr. Ahkami's research group, Dr. Balasubramanian studies different combinations of abiotic stresses in poplar leaves and roots. Each cell type in plant tissue is defined by specific molecular profiles that determine its response to stress. Using integrated spatially resolved single-cell omics, the goals of Dr. Balasubramanian's work are to unravel new elements of plant responses to single and multiple stresses and to map molecular machineries to cellular domains. From investigations of poplar leaf and root cell types, leaf palisade and vascular cells have emerged as exhibiting unique cell-type-specific gene expression patterns under stress. In addition, the combined effect of salinity and heat revealed possible roles of the enzymes zeaxanthin epoxidase and galactinol synthase in specific poplar cell types. Although these enzymes have been linked to heat stress tolerance in previous whole tissue-based studies, using a spatial approach made it possible to identify distinct upregulations in particular cell types.

Further work applied the nanoPOTS (nanodroplet processing in one pot for trace samples) platform for cell-type-specific proteomics analysis of a small number of plant cells. This approach revealed leaf and root cell-type-unique and -shared proteins. Using the optimized nanoPOTS pipeline, Dr. Balasubramanian and his colleagues identified unique candidate cell-type-specific stress-responsive proteins. At the cellular level, a discordance emerged between the transcript and protein abundance data. This finding could suggest a critical layer of regulatory processes at the post-translational level, cell-type-specific variations in splicing patterns, or factors such as mRNA export or protein

stability. Finally, mass spectrometry imaging can enable spatiotemporal metabolite visualization.

Key takeaway: The combination of cell-type transcriptome, proteomic, and metabolic technologies can reveal meaningful information to elucidate cell-type-specific stress responses in plants.

3. **Identification of cell-type specific gene regulatory networks in plants using MINI-EX, a Motif-Informed Network Inference method based on single-cell EXpression data**
   Speaker: **Camilla Ferrari**, VIB-Ghent University, Belgium

Single-cell transcriptomics is a growing goldmine in plants, allowing gene expression levels to be measured in different cell-types. However, single-cell transcriptomics is not sufficient for studying cell-type-specific organization. Dr. Ferrari couples single-cell transcriptomics with gene regulatory network inference through a newly developed method called MINI-EX. This method uses gene expression and transcription factor binding information to infer cell-type-specific gene regulatory networks. MINI-EX is currently available for three plant species: *Zea mays, Oryza sativa,* and *Arabidopsis thaliana.* MINI-EX is executed in three steps: (1) expression-based network inference, (2) transcription factor binding site enrichment, and (3) target gene and transcription factor expression filtering. Compared with existing gold-standard methods (i.e., GRNBoost2 and SCENIC), MINI-EX achieved higher performance metrics.

By applying MINI-EX on an Arabidopsis root single-cell RNA sequencing dataset composed of ~15,000 cells, ~19,000 expressed genes, 41 cell clusters, and 14 cell types Dr. Ferrari identified ~4,000 regulons for ~700 transcription factors. With such a large number of regulons, the next goal was to determine how to correctly prioritize transcription factors relevant for the regulation of a specific organ or condition. For this aim, Dr. Ferrari used a weighted metric that considers the specificity of expression, the importance of the transcription factor for the network of the associated cell-type, and the functional relevance of the target genes. With this metric, MINI-EX was able to identify known root regulators, providing a proof of concept. In addition, MINI-EX identified novel transcription factors linked to root-regulated phenotypes that are likely to be primary regulators.

Key takeaway: Specifically designed for plants, MINI-EX integrates single-cell gene expression and transcription factor motif information to infer cell-type-specific gene regulatory networks and efficiently prioritizes promising candidates for functional characterization studies.

Oral Session 5

1. **Integrating spatial transcriptomics and 3D imaging in plants**
   Speaker: **Kevin Cox, Jr.**, Donald Danforth Plant Science Center, USA

For plants to regulate development and responses to abiotic or biotic stresses, they must communicate. Cell-to-cell communication begins with environmental stimuli, which then triggers inter- and intra-cellular signaling. One key communication means is transcriptional regulation or gene expression. RNA-sequencing is useful for transcriptional analysis in plants, but is limited when performed on bulk tissue, as it fails to resolve spatial and temporal heterogeneity. Spatial transcriptomics is a relatively new technology that allows the preparation of RNA-sequencing libraries from tissue sections, while retaining spatial information.

Within Dr. Meyers' group, Dr. Cox has worked toward increasing the resolution of spatial transcriptomics to a single-cell level by applying maskless array synthesis via photolithography. To reduce the sequence error rate of the microarrays, Dr. Cox shortened the length synthesized on the array. In preliminary experiments, the new high-resolution microarrays for spatial transcriptomics successfully captured mRNA in *Wolffia microscopica*. The next step will be to further optimize the method for RNA-sequencing. In addition, Dr. Cox demonstrated that X-ray microscopy can be used to generate detailed 3D volume image data in *Wolffia microscopica*. Thus, the combination of spatial transcriptomics and 3D imaging provides a powerful new approach for illustrating gene expression localization. Ultimately, this method could elucidate plant–microbe interactions at a single-cell resolution.

Key takeaway: The combination of spatial transcriptomics and 3D imaging technologies can better define transcript abundance patterns across complex plant tissues and organs in a multi-dimensional context.

2. **Mapping plant-mycorrhizal interactions with spatial transcriptomics and single-nuclei sequencing**
   Speaker: **Margot Bezrutczyk**, Lawrence Berkeley National Lab, USA

Symbiosis between plants and arbuscular mycorrhizal fungi (AMF) is critical for agriculture. Plants provide AMF with carbon, and in exchange, AMF provide the plant with phosphorous, nitrogen, and water, partly by acting as a physical extension of the plant's root system. The expected density and yield of modern crops are impossible to achieve without a supplemental phosphate chemical fertilizer. It is expected that we will reach peak phosphorus mining within this decade. Thus, a better understanding of biotic sources of phosphates is critical for the future of agriculture.

During mutualistic symbiosis, AMF grow extensively within the root, both in between and inside the cells. The hypha of the fungus differentiates to form a highly branched structure called an arbuscule. The development of this structure requires high levels of transcriptional changes. Dr. Bezrutczyk and her colleagues study these changes using single-nuclei sequencing and spatial transcriptomics. With *Medicago truncatula* as a

plant model and *Rhizophagus irregularis* as the AMF, single-nucleus sequencing has revealed a variety of cell types, cell states, and new marker genes.

Medicago *gint1* mutants, which fail to form arbuscules, present an ideal model for studying different stages of symbiosis formation. By comparing the transcriptomes of wild-type and *gint1* Medicago*,* Dr. Bezrutczyk is working to delineate the stages of arbuscule formation. Finally, future work on spatial Medicago root-AMF transcriptomics will clarify the location of these events.

Key takeaway: Understanding the symbiosis between plants and AMF is necessary for the future of agriculture. Combining single-nuclei sequencing and spatial transcriptomics will provide a more complete picture of this interaction, including individual cell types at different stages of arbuscule formation.

3. **Plant response to "friend or foe": a cell-type-specific proteomics approach**
   Speaker: **Maite Colinas,** Max Planck Institute for Chemical Ecology, Germany

The Arabidopsis root shows many cell-type-specific biotic responses. When investigating these responses, single cell transcriptomics can be challenging due to potential bias caused by protoplasting in experiments with biotic interactions. Moreover, protein-level assessments are useful because they can account for protein cell-to-cell mobility and subcellular localization. Thus, Dr. Colinas applies cell-type-specific nuclear proteomics via protein proximity labeling. This method is based on the biotinylation of interactors when a biotin ligase is fused to a protein of interest or the biotinylation of all proteins in a subcellular compartment when expressed on its own. A version called Turbo allows proximity labeling at room temperature, which has only recently become available.

In whole seedlings, Dr. Colinas has performed preliminary quantitative nuclear proteomics experiments. As a proof of concept, Dr. Colinas detected early stages of the response to the defense hormone jasmonate, demonstrating the feasibility of this approach. By expressing Turbo under the control of different cell type specific promoters, she is now preparing for cell-type-specific proximity labeling experiments. In future experiments she plans to use this method to determine whether a plant responds differently to pathogenic versus beneficial microbes. This approach may aid in answering how cell-type-specific environmental responses are regulated. Moreover, cell-type-specific proteomics data can also be combined with cell-type-specific metabolomics.

Key takeaways: Protein proximity labeling offers possibilities for identifying regulator candidates at the protein level without tissue disruption and is suitable for quantitative proteomics in response to environmental factors.

Oral Session 6

1. **The plant endoplasmic reticulum at the interface with other organelles: How and what for?**
   Speaker: **Federica Brandizzi,** Michigan State University, USA

Dr. Brandizzi's lab focuses on understanding how and why the plant endoplasmic reticulum (ER) interfaces with other organelles. The ER is at the core of the cell's protein factory and is critical for physiological growth as well as responses to abiotic stress and pathogen attack. The plant ER connects with structures in the cell such as actin and heterotypic membranes. Dr. Brandizzi's lab has defined the proteins involved in some of these interactions. For instance, the SNARE protein SYP73 anchors the ER to the actin cytoskeleton.

In plant cells, the ER is highly motile, yet it is anchored to the plasma membrane. To probe this interaction, Dr. Brandizzi's lab performed *in vivo* pull-down assays and yeast two-hybrid analysis and found that the vesicle-associated protein VAP27-1 interacts with clathrin. Confocal microscopy demonstrated that clathrin is in close association with plasma membrane-ER contact sites. Moreover, endocytosis is partially disrupted in the *vap27-1/3* mutant in plants, as visualized by FM4-64 internalization. This mutant also shows a delay in the formation of clathrin-coated endocytic vesicles, which could be due to a delay in the recruitment of clathrin toward the plasma membrane. Indeed, VAP27 proteins appear to recruit clathrin onto endocytic membranes. Thus, the ER contributes to the function of heterotypic membranes.

The ER network shape changes during cell development. This process requires the ER fusogen root hair defective 3 (RHD3). Loss of RHD3 can enable studies on the effects of a disrupted ER network shape. Interestingly, *rhd3* mutation compromises the movement of non-ER organelles, such as the Golgi apparatus, mitochondria, and peroxisomes. For the Golgi apparatus, live imaging shows that the ER is in close association with the Golgi stacks. For endosomes, the loss of RHD3 disrupts endocytosis, indicating that the ER shape is important for the function of these organelles. Finally, the plant ER physically interacts with chloroplasts for the synthesis of important lipids. Using ER proteomics, Dr. Brandizzi's lab has identified components of this interaction in *Arabidopsis.* For instance, the LURE proteins define an ER subdomain in close association with chloroplasts.

Key takeaways: The plant ER network directly contacts the actin cytoskeleton and heterotypic membranes. Efforts are unraveling the machinery responsible at the interface of the ER with other organelles. ER contacts influence the movement, positioning, and function of other organelles and membranes.

2. **Cell-type-specific behaviors contribute to leaf growth variability in *Arabidopsis***
   Speaker: **Constance Le Gloanec,** University of Montreal, Canada

In developing plant organs, a morphogen gradient is present, causing a general gradient of growth in the cells; however, there is variability in the individual cell

behaviors. It has been suggested that this growth variability plays a role in the acquisition of reproducible plant shapes. Therefore, Ms. Le Gloanec (PhD Candidate) is investigating the origin of growth variability.

Focusing on Arabidopsis first leaf cellular growth, Ms. Le Gloanec's work has revealed that local growth variability is highest in the leaf blade region. To determine the basis of this heterogeneity, Ms. Le Gloanec conducted investigations at the cellular level and found that stomata development appears to underlie growth variability. Indeed, the *spch* mutant, defective in the stomata lineage, does not show the high variability of growth observed in the wild type. Moreover, looking at the growth trajectories of different cell types aligned on the cells' differentiation time, the stomata show a distinct peak in growth during differentiation, followed by a reduction after differentiation is complete. By contrast, pavement cells show a slow reduction in growth rate over time after differentiation. Thus, the cell lineages exhibit specific behaviors. These data suggest that organ growth variability is linked to cell type precise developmental trajectories and the timing of differentiation.

Key takeaway: Cell-type-specific behaviors explain leaf growth variability, and current findings suggest that organ growth variability may result from cell-type heterogeneity in differentiation processes.

3. **Development of targeted single-cell analysis and metabolic imaging of plants using high-spatial-resolution laser ablation electrospray ionization mass spectrometry**
   Speaker: **Michael Taylor,** Pacific Northwest National Laboratory, USA

Metabolites present a functional readout of the cellular and molecular programs controlled by the expression of genes and proteins. Unlike traditional bulk omics methodologies, spatial metabolomics provides spatial information about molecular localization. In particular, laser-ablation electrospray ionization (LAESI) combined with mass spectrometry imaging enables evaluations of tissues and cells in a native state with no additives and provides the spatial distribution of molecules in an *in situ* fashion.

For example, with optical targeting of *Allium cepa* epidermal cells, LAESI can ablate individual cells to obtain a sequential metabolic profile. This approach can also be applied for single-cell analysis across cell populations to reveal metabolic profile differences. One challenge with mass spectrometry is assigning a structure to metabolites based simply on mass. These assignments require an orthogonal measurement. An ion mobility separation system combined with mass spectrometry can trap ions and separate them by size, providing two data points (i.e., size and mass) to determine structure. Finally, epifluorescence imaging can enable optically targeted ablation with LAESI. Future work will refine this technology.

Key takeaway: LAESI is a powerful spatial mass spectrometry technique for metabolomics, enabling native state analysis with little to no molecule degradation, high-throughput single-cell metabolomics, and high-resolution multimodal imaging.

Appendix S3: Community Discussion Padlet Responses

Community responses are ordered based on the number of upvotes received. These comments are intentionally unedited from the Padlet to retain the original input from the community.

Community Discussion 1

What data analysis tools and annotation standards are needed to support and centralize the community's research?

- Similar to consistent ontologies: identifying cell type marker homologues (with open expert curation to identify exceptions) across species would help transfer information between experimental systems **(20 upvotes)**
- Developing a repository where plant and their specific cell type are listed and the data(transcriptomics, proteomics, metabolomics, tissue markers) can be deposited/accessed for the community purpose to compare with non-model organisms. **(18 upvotes)**
- Develop and update -omics comparative tools between plant species to develop more "universal" annotation standards. **(12 upvotes)**
- For standardizing data and providing FAIR metadata...For single cell and spatial transcriptomics- what are the ontologies that people are using to describe their data or what ontologies do we need? **(10 upvotes)**
- Version controls for annotations - Can we establish a platform for the community-driven open-access gene function curation, etc.? esp. for plant biology community? **(9 upvotes)**
- Standards: standard data exchange and file formats... **(7 upvotes)**
- we should also establish more plant species-specific annotation **(7 upvotes)**
- How do we know what is "standard" as tools are constantly being developed and improved? Are any of these tools being developed for plants specifically? **(6 upvotes)**
- Data Analysis Tools: would be good to have a sandbox-type environment that enables trying out all standard and user-uploaded analysis tools as they become available Standards: Need to include all relevant metadata and annotations for published studies! **(6 upvotes)**
- I imagine extensions of this would be needed for single-cell data, but Minimum Information about any (x) Sequence (MIxS) could be a good starting place for experimental metadata: https://gensc.org/mixs/ **(5 upvotes)**
- A gold-standard multimodal dataset for analysis method development and benchmarking **(5 upvotes)**
- Collated cell type-specific expression profiles (or other -omic profiles) for all published studies **(5 upvotes)**
- For single cell (and spatial transcriptomics) data - how are cell types identified? Are these standard across publications? How can these be translated between species? **(5 upvotes)**
- Standardization of methods and data processing, such as sequence data processing or even more difficult, standards for proteomic data, especially for quality control. **(4 upvotes)**
- An Ontology could be developed to describe environmental status of plants profiled in these single cell experiments as more experiments are starting to involve environmental perturbations. **(4 upvotes)**

- How can single cell data be integrated in gene discovery platforms such as Knetminer? **(3 upvotes)**
- Need a central place where people can go to find these standards or tools- some things may already exist or there are related efforts (plugging AgBioData) **(2 upvotes)**
- https://www.humancellatlas.org/data-coordination-2/ **(1 upvote)**
- Complete (i.e. fully-sequenced) organelle genomes for all species **(0 upvotes)**

Community Discussion 2

What types of resources, including computational expertise and cyber-infrastructure, will the community need to best advance the science and meet its goals for broader impacts?

- sc-genomics pipelines documented as a notebook with containerized software images! **(16 upvotes)**
- Push forward the training for data analysis, maybe a summer school for plant sc-datasets **(15 upvotes)**
- Marker gene annotation across species - Database of cell type marker genes and their corresponding orthologs/expressologs/matches across species; synteny maps of good markers; repository to record findings if marker genes translate across species. **(12 upvotes)**
- In addition to computational resources, deposit space for sharing experimental protocols (e.g., nuclei isolation) specialized for plant species, tissue types and etc. **(10 upvotes)**
- easy-to-use data visualizations / dashboards for casual data browsing and a repository to find them **(7 upvotes)**
- An online space for plant sc-RNA-seq community q&a/troubleshooting**. (7 upvotes)**
- For bioinformatics - offer hands-on workshops, especially talking about the "hows and whys" for doing certain analyses. For other techniques (isolating cells, tissue prep) - repository of protocols and people to contact for additional advice for troubleshooting **(7 upvotes)**
- Datasets and packages compatible with mainstream analysis packages/pipelines. E.g. BSGenome packages for Signac/Seurat for model/crop species. **(6 upvotes)**
- A centralized and extensible single-cell visualization browser so that each new study doesn't have to create its own. **(6 upvotes)**
- we need much collaborative effort to use the potential of individual labs in dissecting cellular complexity. Its good to list the participating labs and their expertise on the PCA page for colloborations. we perform lots of single-cell work, but functional work on identified candidates is comparatively slow, that needs to be addressed. **(6 upvotes)**
- Develop methods to integrate scRNA-seq and spatial transcriptomics to assemble different cell types into 3D/4D models. **(5 upvotes)**
- Identify or create Gold Star datasets for teaching / new method development **(4 upvotes)**
- Database listing specific computational expertises of members of the community so they can be contacted by other members needing assistance / input for their analyses **(4 upvotes)**
- accessible training materials distributed freely to the community. Not everyone can attend a physical workshop so consider ways to distribute that are accessible to all. **(3 upvotes)**
- increased social media presence for the public to get excited about plant cells **(2 upvotes)**
- Undergraduate, High school, K-12 education outreach which integrates plant cell genomics **(2 upvotes)**
- list of reporter lines in one place that can help in cell type/cluster annotation process for scRNA-seq data? **(1 upvote)**

- leverage HCA: https://www.humancellatlas.org/data-coordination-2/ **(0 upvotes)**
- Leverage https://www.humancellatlas.org/data-coordination-2/ infrastructure? **(0 upvotes)**

Community Discussion 3

What novel modes of organization and engagement will the community explore to catalyze new ideas, research directions, and discoveries in a time of rapid change?

- Using the PCA slack channels, we may start organizing monthly (or bi-monthly) discussion meetings with a specific topics or journal clubs to facilitate discussion and collaborations? **(13 upvotes)**
- Brainstorming Days: Pre-defined 24 hours of asynchronous discussion focused on very specific topics/questions/ideas on a platform like Slack/Discourse etc. **(11 upvotes)**
- Consider ideation platforms like Hype or Yambla. Establish idea caretaker community and idea processing workflow **(5 upvotes)**
- Identify and organize groups doing/interested in the same types of data collection or analysis around common goals, encouraging collaboration **(5 upvotes)**
- More ECR-focused events? conferences/talk series, to build more community amongst grad students/post-docs. **(4 upvotes)**
- Database of interests and datasets being generated to allow people to more easily connect and collaborate. **(3 upvotes)**
- DataCite has an interesting and interactive way of making their development roadmap/idea processing public. **(3 upvotes)**
- Hype/Yambla request - Is there a link that would give some example of Hype/Yambla workflow? And how best to investigate more deeply? **(2 upvotes)**
- The Single Cell Portal (https://singlecell.broadinstitute.org/single_cell) might provide a template for sharing data, analysis pipelines, etc. **(2 upvotes)**
- sub working groups like the HCA **(1 upvote)**
- public padlet **(0 upvotes)**
- Connect with other Atlas-type projects and model after their datastreams **(0 upvotes)**
- public PCA workspace **(0 upvotes)**

Community Discussion 4

How will the community ensure that individuals and groups who are not regular participants due to disciplinary barriers, cultural differences, or resource limitations are included in the PCA?

- Much of the research is very high cost. To make it most available to the community (including people who may be excluded from data generation), having high expectations for metadata sharing, data sharing and code sharing will help to democratize. **(10 upvotes)**
- Easy access to data sets will be important too, so efforts like EBI's scAtlas will be important **(10 upvotes)**
- more virtual events **(8 upvotes)**
- virtual Research Experience for Undergraduates (REU) program for single cell biology **(5 upvotes)**
- If those individuals still part of plant community (breeders), we can have them in PCA by focusing on proposals on single cell omics on economically important traits of commercially grown varieties (such as disease tolerance, stress tolerance, apart from basic science aspect of cell biology)? **(4 upvotes)**
- Communicating the types of questions that can be addressed with the technology - still lots of talk of "this is neat, but who cares". **(4 upvotes)**

- promote / foster conversations and encouragements to contribute, talk about "imposter syndrome" **(4 upvotes)**
- For the general public: increased social media presence -- Maybe an Instagram plant cell photo contest? **(4 upvotes)**
- clear code of conduct and no tolerance on harassments **(4 upvotes)**
- invent cheaper ways of doing research **(3 upvotes)**
- basic bioinformatics workshops for underserved high school or college students **(3 upvotes)**
- more basic online workshops and webinars for high school and college students with no research experience **(3 upvotes)**
- Special planning groups to understand the needs of communities of interest **(3 upvotes)**
- training on disciplinary techniques to scientists outside of the disciplinary **(3 upvotes)**
- create PCA chapters in underserved countries **(2 upvotes)**
- grants to fund PCA types projects - doing all these projects are expensive, especially the sequencing! **(2 upvotes)**
- Get undergrads to produce content for social media: tik tok, instagram, twitter **(2 upvotes)**
- promote DIY in plant cell research **(1 upvote)**
- promote PCA to rural america **(1 upvote)**
- Create a panel of experts in the PCA community who could serve as mentors/advisors that people in smaller countries, rural colleges, etc. could go to for advice. **(1 upvote)**
- Virtual events with more natural networking/discussion options (e.g. Gather.town has a easy interface for meeting people that emulates real conference experience of "walking into someone") **(0 upvotes)**
- There are some interesting imaging DIY info out there, I don't it has been aggregated **(0 upvotes)**
- A channel that redistribute latest development news directly from the researcher desks to all the interested audience (e.g. students, other labs) on multiple social media channels. **(0 upvotes)**

Community Discussion 5

In what ways will the community recruit, train, and nurture new talent to seed paradigm-shifting discoveries in the future?

- Bringing onboard people from other disciplines, entertaining ideas of diverse natures, enabling them through ready to engage platforms/ collaborator teams/ events. **(9 upvotes)**
- Teach plant sciences (PCA) in bioinformatics/IT/compsci programs and bioinformatics in plant bio programs (at BSc level)! **(5 upvotes)**
- These technologies are exciting and powerful, but expensive and specialized. Access and funding are really key. **(4 upvotes)**
- Gold star datasets and open challenges on Kaggle or other data repositories **(3 upvotes)**
- EMSL user programs is a good way to have students visit from the partnering lab and learn about the new technologies. **(3 upvotes)**
- Leveraging the ease of experimentation in plant biology as a testbed for specific questions in human biology/disease, creating cross disciplinary collaboration and taking share in budgets from NIH, **(3 upvotes)**
- Lobby for funding initiatives from public and private sources to develop and/or adapt new tech for plant tissue **(3 upvotes)**

- Expose students K-12 to gardening and emerging technologies to grow plants like vertical farming, hydroponics, shipping container farming. Make them curious first and then hit them with all these open questions they can contribute **(3 upvotes)**
- maybe with hackaton contest **(3 upvotes)**
- Develop educational materials for easy additions to core graduate training: conceptual, hands-on, etc **(3 upvotes)**
- Host plant science competitions with an award at the end. E.g. seek proposals for studies in plants in space and the winner/s get their project sent by SpaceX **(2 upvotes)**
- Really exciting presence on social media for non-scientists (or... not yet scientists) **(2 upvotes)**
- teach plant biology at engineering schools **(2 upvotes)**
- PCA developed fliers for exciting plant science technology needs for distributiuon to Engineering departments **(2 upvotes)**
- Coordinate internships for graduate students at 10x genomics, Resolve, other popular commercial solutions providers **(2 upvotes)**
- Sharing tools, creating/setting up collaborations with people not in "typical" fields of associated with PCA (e.g. evolution, ecology) **(1 upvote)**
- Open textbooks or periodic reviews articles to outline state-of-the-art technologies **(1 upvote)**
- Undergraduate career path materials for increased awareness of jobs in plant sciences **(1 upvote)**
- teach how to explore the unknown as well as what's already known **(1 upvote)**
- More training workshops to get people familiar with the single-cell datasets. Encourage people to use single-cell datasets and promote funding in the area that can attract more people to work on this area. **(1 upvote)**
- As discussed, bringing in students and talent form outside plant science is critical. Can more be done to identify the grand challenges (and underlying motivations) to departments that attract this talent - engineering, physics, computation, chemistry etc. **(0 upvotes)**
- For new tech development, how could the PCA help build bridges between engineering labs and departments and plant scientists? **(0 upvotes)**
- Influence major textbook authors to get plant cell science in core undergraduate education **(0 upvotes)**

Community Discussion 6

How will the outcomes of the community's efforts serve the wider scientific community?

- We need to convince the wider scientific community of the value of single-cell approach to 1- better understand plant biology; 2- use of this knowledge to design new strategies to improve crop performance. **(3 upvotes)**
- go beyond plants, inter-kingdom communities **(2 upvotes)**
- Bringing more attention to plants as essential tools for understanding cell biology in general by building community and focus, teaming up to get the attention of animal biologists and those in industry **(2 upvotes)**
- state / land grant universities have outreach offices (extension) that will connect you directly with farmers. can we leverage this opportunity? **(2 upvotes)**
- Use GRC to bring the disparate communities together **(2 upvotes)**
- education community--high school teachers **(2 upvotes)**
- how can the single-cell community come together with the traditional plant bio community? **(2 upvotes)**

- the results can help to understand other biological mechanisms in other organisms **(2 upvotes)**
- sustainable, long-term place where data can be made as accessible as possible for people who are non-experts **(2 upvotes)**
- AGBT conference would be a great venue to connect PCA with the wider community **(1 upvote)**
- Try to influence funders to put resources into one sustainable database to house the data **(1 upvote)**
- promote the differences between cell types and individual cells within a cell type that might not been appreciated before **(1 upvote)**
- invite these 'outside' stakeholders, wider research community members to the GRC **(0 upvotes)**
- how to engage industry, breeders, farmers **(0 upvotes)**

Appendix S4: Feedback Survey Responses

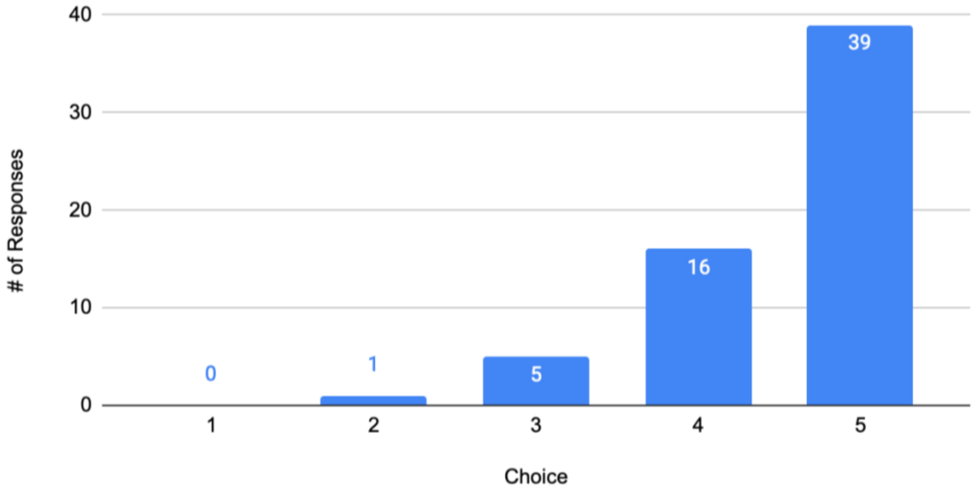

Figure S1: How relevant and helpful was the 2021 PCA Symposium for your research? Results from survey question #1 (N=61 responses). The question was posed with a 5-point likert scale ranging from 1 (Not Helpful) to 5 (Very Helpful). 55 respondents reported a rank of 4 or 5 (~90%).

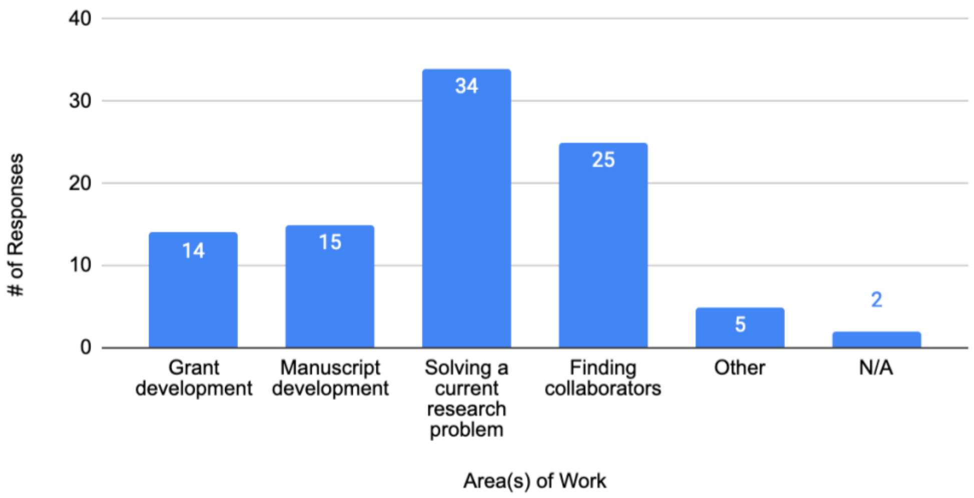

Figure S2: Which areas of your work was this symposium helpful for, if any? Results from survey question #2 (N=57 responses, but 27 respondents chose more than one area).

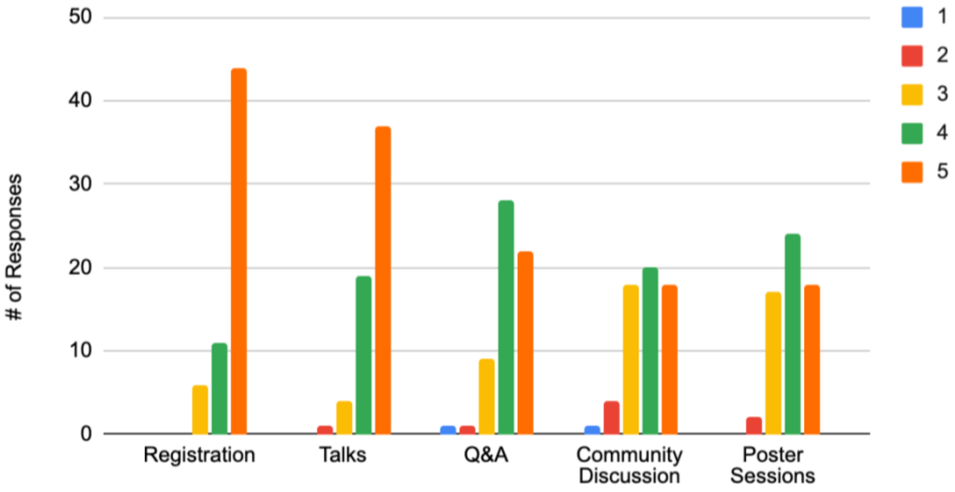

Figure S3: How satisfied were you with the following workshop components? Results from survey question #3 (N=61 responses). The question was posed with a 5-point likert scale ranging from 1 (Very Dissatisfied) to 5 (Very Satisfied).

Table S1. Any overall feedback for the event (what worked well and what didn't work well)? Results from survey question 4 (N=32 responses). We coded the responses into 3 categories: General Positive Response, Constructive Criticism, and General Criticism. We then identified 6 subcategories of Constructive Criticism comments: poster sessions (3), community discussions (4), networking opportunities (2), talk titles in accelevents (4), time zone (2), and other (4). These comments are intentionally unedited from the survey to retain the original input from the community.

Comment	Category
everything is good	**General Positive Response**
It is really good	General Positive Response
Overall it's a wonderful symposium	General Positive Response
It was great	General Positive Response
its great symposium,	General Positive Response
I like this symposium. I learned a lot. The presenter answering the question through the chat is super nice!	General Positive Response
Well organized.	General Positive Response
Talks and poster work well.	General Positive Response
This meeting was one of my 2021 highlights. I really enjoyed the talks - almost all were excellent. I also enjoyed the "working" sessions where we discussed the future of our field. I want to have more of these discussions, they were collegial and reminded me of how bright and thoughtful many of our peers are. Also,	General Positive Response

I think they were productive. Thanks.	
I think the small poster sessions in this symposium worked better than the open structure that other virtual conferences have used, but still can't compare to a real poster session. Hopefully next year will be in person!	***Constructive Criticism: Poster Sessions***
arrange a flash talk slot for the poster section.	*Constructive Criticism: Poster Sessions*
everything was very smooth, really liked having 2-3 concurrent posters. they could be made into concurrent talks in the future rather than posters, since that might work better in a zoom setting. i think poster is good for in person experience but not for the online one.	*Constructive Criticism: Poster Sessions*
Most of the contributed talks were excellent. I found the level of the invited/keynote speakers way more variable (for some it was not clear to me how they fitted in the PCA theme). The Community Discussion was not very fruitful and sometimes disappointing, as a lack of expertise and well-informed opinions resulted in superficial comments, without a clear path how to turn these in concrete plans or action points. Is the goal to have follow-up meetings about points raised? Thanks for organizing this great event!	***Constructive Criticism: Community Discussions***
I did not find the community discussion of high level. Clearly, many people that posted questions were not well prepared (e.g. we don't need new ontologies, please explore the available (plant) ontologies or suggest modifications, if needed). A panel of experts, preparing key questions, could have delivered a more in-depth overview of outstanding questions and solutions. The presentations and poster sessions were excellent.	*Constructive Criticism: Community Discussions*
Can't answer questions for posters because i did not go- should have N/A option. The padlet format of the discussion was weird- I thought previous organization with breakout rooms and discussion leader was better because it seemed to encourage more participation. The padlet could be used in those and shared back to the group but I think there would have been more equitable discourse in smaller groups.	*Constructive Criticism: Community Discussions*

"There were a lot of community discussion sections - which were also great breaks! But these discussions appeared to only involve a fraction of the people - is there a way to get more people involved as well? Did we need so many of these discussions? Poster sessions were fine - I was a bit too shy to go and meet the people, but it was nice to see their posters."	*Constructive Criticism: Community Discussions*
The organizers made a big effort and pulled together an interesting conference. Technically everything worked well. Speakers and moderators were mostly well chosen (only in one case a moderator's comment was borderline rude). The community sessions worked well but seemed to be more useful for the organizers than for the participants. It would be good to promote more direct interactions between participants including ECRs for the next symposium. I understand it is not easy in a virtual format but there are concepts like virtual dinner tables for example. In its current format there was no chance to directly interact with others or engage in conversations.	***Constructive Criticism: Networking Opportunities***
More interaction with other participants would have been nice (networking/discussion "tables" or zoom rooms)	*Constructive Criticism: Networking Opportunities*
Next time, please be sure to include the title of the presentation immediately adjacent to each speakers name with Accelevents. It was very difficult to know what each speaker was going to cover without going back and forth between different documents.	***Constructive Criticism: Talk Titles in Accelevents***
It is better to have the talk titles displayed next to the names of speakers.	*Constructive Criticism: Talk Titles in Accelevents*
It was impossible to find talk title or topic in the agenda. I wish I could have found it in the agenda or pdf Symposium program. It is not very informative having only presenters' name and no talk title.	*Constructive Criticism: Talk Titles in Accelevents*
It would be great if we can see the title of the talk of each speaker in the schedule at the main stage room or the front desk.	*Constructive Criticism: Talk Titles in Accelevents*
The format and platform worked. Personally, the time difference didn't work for me, but still a great event! Amazing job everyone!	***Constructive Criticism: Time Zone***

Great symposium. The only issue I had was the early start time on the west coast so I had to miss several live talks. But of course there is no way to make it convenient for everyone, and it was great that the talks were recorded and could be watched later.	*Constructive Criticism: Time Zone*
Really great talks! Feedback I got from colleagues is that they were surprised a bit by the topics (scRNA- and spatial transcriptiomics heavy) because in earlier adverts it sounded more metabolism focused. They said they would have submitted posters to an scRNA theme. So perhaps in the future, make it more clear ahead of time what the focus will be.	***Constructive Criticism: Other***
Registration was a bit confusing but was able to figure it out (had to "buy ticket" before entering the event)	*Constructive Criticism: Other*
"Everything was OK.	*Constructive Criticism: Other*
As a person working with 'common' gel-free proteomics and metabolomics, I am impressed with the methodological advances in plant research in recent years. I hope to establish scientific cooperation with people who perform spatial metabolomics / transcriptomics analyzes (I will start my search with people presenting the results at the PCA symposium!). So, my only remark may be: too few people not directly involved in these advanced analyses know about PCA."	*Constructive Criticism: Other*
Could not join community discussions	**General Criticism**
lecture should be available on youtube after session over	General Criticism
There were two chats, q&a and chat, it was confusing, but it was solved.	General Criticism
Presentawork well..discussion rounds little upset me	General Criticism

Table S2. What are the types of technologies you would like to hear about in a future PCA webinar? Results from survey question 5 (N=24 responses). We coded the responses into 4 categories: single cell omics (10), data analysis (4), imaging (5), and other (5). These comments are intentionally unedited from the survey to retain the original input from the community.

Comment	Category
single cell genomics	**Single Cell Omics**

single cell sequencing related wet and dry techniques, imaging techniques	Single Cell Omics
Single Cell Technology	Single Cell Omics
single-cell data analysis; spatial transcriptomics-based hypothesis generation	Single Cell Omics
single-cell sequencing	Single Cell Omics
Spatial transcriptome and high-resolution imaging	Single Cell Omics
Spatial Transcriptomics	Single Cell Omics
Spatial transcriptomics	Single Cell Omics
All the time: spatial OMICs.	Single Cell Omics
It would be nice to hear about a wider variety of technologies. There was little to no single cell metabolomics or proteomics, almost all talks were about single cell sequencing or spatial transcriptomics.	Single Cell Omics
I'd like to see more information on analysis - beyond how to do the basics in Seurat, for example. What can we actually do with some of these data?	**Data Analysis**
Guidance/best practices for analyzing single cell data (and pitfalls); spatial transcriptomics how to (development of the right section/"chip" to the analysis)	Data Analysis
more bioinformatics tools and solutions	Data Analysis
In addition to research tech (spatial transcriotmics), I'm really curious of the tools/programs to support collaborative idea generation	Data Analysis
image segmentation automation	**Imaging**
Imaging Analysis freeware and open source applications	Imaging
Imaging of plant cells under stress conditions.	Imaging
Cell Atlas imaging	Imaging
I'd be interested in hearing talks about expansion microscopy, new techniques in spatial transcriptomics, and cryo-EM	Imaging
13C metabolic flux analysis	**Other**
Base editing	Other
Effect of protoplasting on scRNA-seq	Other
NA	Other
Quantitative chromatin analysis	Other

Appendix S5: Symposium Participants

Across the sessions, nearly 500 scientists participated, of which about 60% were early-career researchers (i.e., graduate students, postdoctoral fellows, or assistant faculty) and about 40% were non-US based. A total of 48 countries were represented by the attendees.

List of Participants:
Brad Abramson, Noblis
Nathan Abundo Tran, University of California Los Angeles
Johanna Acevedo-Garcia, KeyGene
Kelvin Adema, Wageningen University and Research
Muhammad Adil, Seoul National University
Pinky Agarwal, National Institute of Plant Genome Research
Amir H. Ahkami, Environmental Molecular Sciences Laboratory, Pacific Northwest National Laboratory
Sahar Alshareef, Jeddah University
Vivek Ambastha, Migal Galilee Research Institute
Gazala Ameen, South Dakota State University
Stig U. Andersen, Aarhus University
Christopher Anderton, Pacific Northwest National Laboratory
Davide Annese, University of Cambridge
Alejandro Aragon, North Carolina State University
Jodie Armand, University of Sheffield
Arif Ashraf, University of Massachusetts Amherst
Kyaw Aung, Iowa State University
Femke Auwelaert, Ghent University
Anna Backhaus, John Innes Centre
Ahmet Bakirbas, University of Massachusetts, Amherst
Vimal Kumar Balasubramanian, Pacific Northwest National laboratory
Martina Balboni, University of Hamburg
Ali Mohammad Banaei-Moghaddam, University of Tehran
Bastiaan Bargmann, Virginia Polytechnic Institute and State University
Rachel Baschieri, Oregon State University
Tobias Baskin, University of Massachusetts
Suvajit Basu, Banaras Hindu University
Vijaya Battala, Cornell University
Kevin Bellande, Université de Neuchâtel, Neuchâtel
Etienne Bellani, University of Lausanne
Tanya Berardini, The Arabidopsis Information Resource/Phoenix Bioinformatics
Dominique Bergmann, Stanford University
Judith Bernal, CINVESTAV
Margot Bezrutczyk, Lawrence Berkeley National Lab
Kaushal Bhati, Louvain Institute of Biomolecular Sciences, Université Catholique de Louvain
Payel Bhattacharjee, Norwegian University of Life science

Samik Bhattacharya, Resolve Biosciences
Filippo Binci, University of Padova
Kenneth Birnbaum, New York University
Subhankar Biswas, Banaras Hindu University
Noel Blanco, ETH Zurich
Thomas Blein, IPS2/CNRS
Norbert Bollier, French National Institute for Agriculture, Food, and Environment
Ignacy Bonter, University Of Cambridge
Rita Borna, University of Dhaka
Alex Borowsky, University of California Riverside
Elvis Branchini, Université de Montréal
Susanne Brink, Trends in Plant Science / Cell Press
Matthew Brooks, USDA-ARS/UIUC
Jennifer Brophy, Stanford University
Javier Brumos, Institute for Plant Molecular and Cell Biology
Haoran Cai, Massachusetts Institute of Technology
José Carballo, CERZOS-CONICET
Alan Cervantes-Perez, LANGEBIO-CINVESTAV
Sonhita Chakraborty, N/A
Mingqin Chang, University of California Davis
Ajeet Chaudhary, Plant Biology Carnegie Science
Rim Chaudhury, Presidency University, Kolkata
Sixue Chen, University of Florida
Hongyu Chen, Zhejiang University
Liangliang Chen, HHMI
George Chuck, University of California Berkeley
Steve Clouse, National Science Foundation
Gitta Coaker, University of California Davis
Jeremy Coate, Reed College
Benjamin Cole, DOE-Joint Genome Institute
Maite Colinas, Max Planck Institute for Chemical Ecology
Lee Conneely, La Trobe University
Stephanie Conway, Harvard University
Sandra Correia, University of Coimbra
Danny Cowan-Turner, Newcastle University
Kevin Cox, Donald Danforth Plant Science Center
Brian Crawford, Pairwise
Nathalie Crepin, KULeuven
Charles Cunningham, National Science Foundation
Josh Cuperus, University of Washington
Kirk Czymmek, Donald Danforth Plant Science Center
Jeevitha D, N/A
Jaishri Rubina Das, National Institute of Plant Genome Research, New Delhi, India
Kirtan Dave, GSFC University
Lisa David, North Carolina State University

Stefan de Folter, UGA-Langebio, CINVESTAV
Koen De Gelas, 10x Genomics
Daniela De la Mora Franco, LANGEBIO
Ylenia De Luca, University Of Naples Federico II
Natalia de Val, Thermo Fisher Scientific
Edgar Demesa-Arevalo, Heinrich-Heine University
Peter Denolf, BASF - BBCC Innovation Center Gent
Tom Denyer, ZMBP, University of Tuebingen
Thomas Depuydt, VIB-UGent Center for Plant Systems Biology
Kshitiz Dhakal, Virginia Polytechnic Institute and State University
Diane Dickel, Lawrence Berkeley National Lab
Steve DiFazio, National Science Foundation
Peter Dimba, Freiburg University
Pingtao Ding, Institute of Biology Leiden, Leiden University
Xue Ding, Rutgers University
Indeewari Dissanayake, VIB-UGent Center for Plant Systems Biology
Anika Dolata, Heinrich-Heine-University Düsseldorf
Douglas Domingues, UNESP, São Paulo State University
Juan Dong, Waksman Institute
Georgia Drakakaki, University of California Davis
Gavin Duggan, Alphabet Inc.
Thomas Eekhout, VIB Center for Plant Systems Biology
David Ehrhardt, Carnegie Institution for Science
Aurélia Emonet, Max Planck Institute for Plant Breeding Research
Tara Enders, Hofstra University
Isil Erbasol Serbes, University of Bremen
Jose Estevez, Fundación Instituto Leloir (Argentina) and UNAB-iBIO (Chile)
Kellye Eversole, International Wheat Genome Sequencing Consortium
Michelle Facette, University of Massachusetts, Amherst
Noah Fahlgren, Donald Danforth Plant Science Center
Yves Falanga, N/A
Mehdi Fazel, Oklahoma Medical Research Foundation
Lavinia Ioana Fechete, Aarhus University
Lanlan Feng, The Chinese University of Hong Kong
Song Feng, Pacific Northwest National Laboratory
Rebeca Fernandez Lara, Oterra
Camilla Ferrari, VIB-UGent Center for Plant Systems Biology
Sterling Field, Carnegie Institution of Science
John Fowler, Oregon State University
Margaret Frank, Cornell University
Manuel Frank, Aarhus University
Lamprinos Frantzeskakis, Bayer CropScience
Gaurav Gajurel, Arkansas State University
Teresa Rosa Galise, University of Naples Federico II
Venerando Gambuzza, University of Catania

Julia Paulina García González, Langebio
Anthony Garza, National Science Foundation
Ashok Gehlot, CSIR - Institute of Himalayan Bioresource Technology
Majid Ghorbani Javid, University of Tehran
Marco Giovannetti, University of Padova
Tatsuaki Goh, Nara Institute of Science and Technology
Camila Goldy, Centre National de la Recherche Scientifique
Fabio Gomez, Michigan State University
Maria Gomez, Centre National de la Recherche Scientifique
Andrea Gomez Felipe, University of Montreal
Yan Gong, Harvard University
Jerry González-Cantoral, University of San Carlos of Guatemala
Theresa Good, National Science Foundation
Sharon Greenblum, Joint Genome Institute
Julien Gronnier, University of Tuebingen
Nan Gu, Utsunomiya University
Bruno Guillotin, New York University
Apoorva Gupta, National Institute of Plant Genome Research, New Delhi
Sofya Gvaramiya, ARRIAB
Susinya Habila, Chulalongkorn University
Maria Harrison, Boyce Thompson Institute
Elizabeth Haswell, Washington University in St. Louis
Ericka Havecker, Bayer Crop Science
Michelle Heeney, Cornell University
Yka Helariutta, University of Cambridge
Hannah Hendrickson, University of Massachusetts Amherst
Venura Herath, University of Peradeniya
Humberto Herrera-Ubaldo, Cinvestav
Maarten Hertog, Katholieke Universiteit Leuven
Manju Hingorani, National Science Foundation
Kim Hixson, Environmental Molecular Sciences Laboratory
Kimmy Ho, IPMB, Academia Sinica
Srinidhi Holalu, University of California Berkeley
Uyen Hong, La Trobe University, Australia
Guojian Hu, Laboratoire de Recherche en Sciences Végétales
Carol Huang, New York University
Lucia Ichino, University of California, Los Angeles
Julio Iehisa, Universidad Nacional de Asuncion
René Inckemann, MPI Marburg for Terrestrial Microbiology
Sessen Daniel Iohannes, Cold Spring Harbor Laboratory
Sandra Irmisch, Leiden University Institute of Biology
Rand Ismaeel, University of Southampton
David Jackson, Cold Spring Harbor Laboratory
Jonathan Jacobs, Ohio State University
Suryatapa Jha, Claremont Colleges

Yinping Jiao, Texas Tech University
Lala Jimenez, PGC
Leonardo Jo, Utrecht University
Sanjay Joshi, University of Kentucky
Kaisa Kajala, Utrecht University
Kuldeep Kandarkar, Tamil Nadu Agricultural University Coimbatore
Hassan Karim, Triticeae Research Institute
Rakkammal Kasinathan, Alagappa University
Ramesh Katam, Florida A&M University
Mather Khan, Heinrich Heine Universität Düsseldorf
Daniel Kierzkowski, University of Montreal
Sunran Kim, Bayer Crop Science
Seong-Ryong kim, Sogang University
Ji-Yun Kim, Heinrich-Heine-Universität Düsseldorf
Minsoo Kim, Galy
Jiyun Kim, Heinrich Heine Universität Düsseldorf
Eun Deok KIM, University of Texas at Austin
Ayaka Kinoshita, National Tax Agenco
Ryan Kirkbride, University of Texas at Austin
Anastasiya Klebanovych, Donald Danforth Plant Science Center
Eva Knoch, LMU
Dae Kwan Ko, Michigan State University
Shuyao Kong, Cornell University
Anne Krapp, French National Institute for Agriculture, Food, and Environment
Chi Kuan, IPMB, Academia Sinica
Pradeep Kumar, Centre for Cellular & Molecular Biology
Pawan Kumar, National Institute Of Plant Genome Research
Pankaj Kumar, Parmar University of Horticulture and Forestry
Ritesh Kumar, University of Minnesota
Sunita Kumari, Cold Spring Harbor Laboratory
Lucia Kusumawati, International University Liaison Indonesia
Jickerson Lado, University of the Philippines Los Baños
Julien lang, French National Institute for Agriculture, Food, and Environment
Dmitry Lapin, Utrecht University
Rahul Lathe, Swedish University of Agricultural Sciences
Constance Le Gloanec, Plant Research Institute - University of Montreal
Jiyoung Lee, Virginia Tech
Young-Jin Lee, Iowa State University
Travis Lee, Salk Institute
Chunghee Lee, Korea University
Samuel Leiboff, Oregon State University
Tedrick Lew, A*STAR
Mathew Lewsey, La Trobe University
Zhongpeng Li, Iowa State University
Dongmeng Li, Waksman Institute of Microbiology, Rutgers University

Liu Li, Ghent University
Mao Li, Donald Danforth Plant Science Center
Chenxin Li, University of Georgia
Song Li, Virginia Tech
Pengbo Liang, Uni Freiburg
Irene Liao, University of California, Berkeley
Marc Libault, University of Nebraska Lincoln
Boon Leong Lim, University of Hong Kong
Shey Li Lim, University of Hong Kong
Chien-Yuan Lin, Lawrence Berkeley National Laboratory
Bouyun Lin, Ghent University
Penelope Lindsay, Cold Spring Harbor Laboratory
Maneesh Lingwan
Su-Ling Liu, Iowa State University
Le Liu, University of Massachusetts, Amherst
Yang Liu, University of British Columbia
Tie Liu, University of Florida
Jinhong Liu, University of Hong Kong
Ao Liu, Stanford University
Eliza Loo, Heinrich-Heine Universität
Jose Lozano Torres, Wageningen University & Research
Danying LU, Zhejiang A&F University
Agnieszka Ludwików, Adam Mickiewicz University in Poznań
Leonie Luginbuehl, University of Cambridge
Valentín Luna García, CINVESTAV - LANGEBIO
Ziliang Luo, University of Florida
Ai-My Luong, Louvain Institute of Biomolecular Sciences, Université Catholique de Louvain
Munan Lyu, University of Helsinki
Meeri Mäkelä, University of Helsinki
Rex Malmstrom, DOE Joint Genome Institute
Kamal Kumar Malukani, CSIR-CCMB
Nicolás Manosalva Pérez, VIB-UGent Center for Plant Systems Biology
Concepcion Manzano, University of California Davis
Olivier Martin, French National Institute for Agriculture, Food, and Environment
Irene Martinez, Umea University
Sarah Mathura, University of the West Indies
Nuria Mauri, Centre for Research in Agricultural Genomics
Conor McClune, Stanford University
Heather McFarlane, University of Toronto
Maria Camila Medina Montes, Universidade de São Paulo
Devang Mehta, University of Alberta
Rachel Melton, N/A
Jonathan Meuser, Chi Botanic Inc.
Heather Meyer, Carnegie Institute of Science
Todd Michael, Salk Institute for Biological Studies

Kari Miller, Washington University in St. Louis
Ya Min, University of Connecticut
Miguel Miñambres Martín, Plant Biochemistry
Javier A Miret, University of Reading
Victoria Mironova, RIBES, Radboud University
Bharat Mishra, University of Alabama at Birmingham
Tasleem Mohammad, ICAR-NIPB
Bruna Montes Luz, University of Missouri
Matheus Montrazi, Université de Bordeaux
William Moore, University of California San Diego
Mahdi Morad Pour, Tallinn University of Technology
Sebastian Moreno, Sainsbury Laboratory
Bethan Morris, Newcastle University
Jenny Mortimer, University of Adelaide
Jose Muino, Humboldt University
Sreeshma N, Indian Agricultural Research Institute
Peter Nagy, University of Kentucky
Akshay Nair, VIB-UGENT
Keiji Nakajima, Nara Institute of Science and Technology
Qiong Nan, University of Massachusetts
Santiago Navarro, Bayer Crop Science
Nazrul Hisham Nazaruddin, MARDI
Nils Nebel, University of Freiburg
Mamta NEHRA, International Centre for Genetic Engineering and Biotechnology
Hilde Nelissen, UGent/VIB
Jason Nichols, Syngenta
Sebastiano Nigris, University of Padova
Luke Nikolov, University of California Los Angeles
Ido Nir, Stanford University
Tatsuya Nobori, Salk Institute for Biological Studies
Trevor Nolan, Duke University
Muhammad Noman, National Institute for Genomics and Advanced Biotechnology
Michael O'Cain, N/A
Lily O'Connor, Donald Danforth Plant Science Center
Ronan O'Malley, DOEJoint Genome Institute
Dong-Ha Oh, Louisiana State University
Odunayo Ojo, Lead City University
Marina Oliva, University of Western Australia
Shohei Oshiro, Nara Institute of science and technology
Zulnaray Osman, University of Tokyo
Bolaji osundahunsi, International Institute of Tropical Agriculture, Nigeria
Marisa Otegui, University of Wisconsin-Madison
Sofia Otero, University of Cambridge
Miroslav Ovecka, Palacky University
Takehiro Ozawa-Uyeda, CINVESTAV Unidad Irapuato

Lingeshwaran P K, Institute Of Forest Genetics And Tree Breeding
Gergo Palfalvi, National Institute for Basic Biology
Xue Pan, University of California Riverside
Aleksandar Parvanov, ZMBP, University of Tubingen
Taras Pasternak, Bio II
Sandi Paulisic, University of Lausanne
Bhausaheb Pawar, Humboldt-Universität zu Berlin
Ullas Pedmale, Cold Spring Harbor Laboratory
Marta Peirats-Llobet, La Trobe University
Imara Perera, North Carolina State University
Emerald Perlas, EMBL-Rome
Anna Pijnacker, WUR
Anne Plant, National Institute of Standards and Technology
Daniil Prigozhin, Lawrence Berkeley National Laboratory
Nicholas Provart, University of Toronto
Gibbs Qian, Zhejiang University
Elsa Herminia Quezada Rodríguez, Universidad Nacional Autónoma de México
Maryam Rafiqi, UM6P
Carin Ragland, Stanford University
Prashanth Ramachandran, Stanford University
Daniel Ramirez, Carnegie Institution for Science
Egon Ranghini, 10x Genomics
Sombir Rao, Cornell University
Lavi Rastogi, Regional Centre for Biotechnology
Ravindra Raut, National Institute of Technology Durgapur
Emma Raven, John Innes Centre
Marius Rebmann, University of Cambridge
Amey Redkar, University of Cordoba
Ahmed Refaiy, University of Leeds
Dugald Reid, Aarhus University
Leonore Reiser, Phoenix Bioinformatics/TAIR
Mauricio Reynoso, IBBM UNLP CONICET
Leila Rezaei Somee, University of Tehran
Sue Rhee, Carnegie Institution for Science
Selena Rice, Carnegie Institution for Science
Laura Righetti, University of Parma
Sean Robertson, University of Manitoba
Marta Rodriguez Franco, Freiburg University
Facundo Romani, University of Cambridge
Maida Romera Branchat, Institute of Plant Biology and Biotechnology
Luis C. Romero, CSIC
Daniele Rosado, Cold Spring Harbor Laboratory
Josh Rosnow, Simplot
Ronelle Roth, University of Oxford
Sandrine Ruffel, French National Institute for Agriculture, Food, and Environment

Diana Ruggiero, Oregon State University - Department of Botany and Plant Pathology
Yue Rui, Stanford University
Rajan Kumar Sah, Regional Center For Biotechnology
Sushree Sangeeta Sahoo, Calcutta University
Solihu Kayode Sakariyahu, University of Manitoba
Mohammad Salehin, University of North Carolina at Greensboro
Lekshmy Sathee, IARI
Bob Schmitz, University of Georgia
Gerald Schoenknecht, National Science Foundation
Sebastian Schornack, University of Cambridge
Juan Pablo Selva, CERZOS
Hyemin Seo, The University of Texas at Austin
Karen Serrano, Joint Bioenergy Institute
Nelson Serre, Charles University
Muhammad Subhan Shafique, University of Agriculture Faisalabad
Rachel Shahan, Duke University
Paras Sharma, CSIR-IHBT
Rahul Shaw, Radboud University
Abbas Shojaee, New York University
Hala Showre, Stanford
Monika Shrivastava, National institute of plant genome research
Meredith Sigman, Bayer
Sylvia Silveira, University of Montreal
Rüdiger Simon, Heinrich Heine University
Rosalie Sinclair, University of California Davis
Anjali Singh, Freelance
Robin Slof, Anthura B.V.
Marija Smokvarska, CNRS
Shyam Solanki, South Dakota State University
Pavel Solansky, University of Tuebingen
Davide Sosso, GoogleX
Fred Souret, 10x Genomics
Gary Stacey, University of Missouri-Columbia
Yvonne Stahl, Heinrich-Heine University
Thomas STANISLAS, ZMBP- Universität Tübingen
Josh Strable, North Carolina State University
Jasper Stroeder, N/A
Chao Su, University of Freiburg
Adriana Suarez, 10x Genomics
Kawinnat Sue-ob, University of Liverpool
Hendry Susila, Korea University
Pablo Szekely, Duke University
Wojciech Szponarski, French National Institute for Agriculture, Food, and Environment
Agnieszka Szuba, ID PAS
Alicia Guadalupe Talavera-Caro, West Virginia State University

Vaijayanti Tamhane, Savitribai Phule Pune University
Huang Tan, Center for Plant Molecular Biology
Bozeng Tang, Sainsbury Laboratory
Hugo Tavares, University of Cambridge
Akshay Tawale, IISER Tirupati
Michael Taylor, Pacific Northwest National Laboratory
Francesca Tedeschi, Aarhus University
Estee Tee, John Innes Centre
Ooi-Kock Teh, Institute of Plant and Microbial Biology, Academia Sinica
Cristovao Teixeira, University of Neuchatel
Ricardo Tejos, Universidad Arturo Prat
Chong Teng, Donald Danforth Plant Science Center
Sutton Tennant, University of Nebraska Lincoln
Philipp Ternes, BASF Metabolome Solutions GmbH
Delia TERRANOVA, University of Naples Federico II
Martha Thellmann, University of Zurich
Xin Tian, Radboud University
Prakash Timilsena, Virginia Tech
Trevor Tivey, Cornell University
Sepideh Torabi, University of Guelph
Keiko Torii, University of Texas At Austin
Tuan Tran, University of South Alabama
Frej Tulin, Carnegie Institution for Science
Senem Ugur, Ornamental Plants
Israr Ud Din, The University of Agriculture Peshawar
Glen Uhrig, University of Alberta
Petra van Bergeijk, KeyGene
Lisa Van den Broeck, North Carolina State University
Maurice van Veen, Anthura Research BV
Mieke van Vlaardingen, Leiden University
Robert VanBuren, Michigan State University
Klaas Vandepoele, Ghent University
Katy Vandereyken, Katholieke Universiteit Leuven
Kendra Vazquez Molina, Bayer Crop Science
Zuzana Vejlupkova, Oregon State University
Tamara Vellosillo, Stanford University
Joop Vermeer, University of Neuchâtel
Inge Verstraeten, CNRS
Carlos Vidal-Céspedes, University of Chile
Marija Vidovic, Institute of Molecular Genetics and Genetic Engineering
Josep Vilarrasa Blasi, Stanford University
Giang Vo, Seoul Nation University
Thierry Voet, KU Leuven
Michelle von Arx, Nano Signaling Lab
James Walker, John Innes Centre

Justin Walley, Iowa State University
Zhiyong Wang, Carnegie Institution for Science
Shanshan Wang, Max Planck Institute for Developmental Biology
Jinzheng (Gene) Wang, University of California Riverside
Guannan Wang, Louisiana State University
Jianping Wang, University of Florida
Binghan Wang, University of Montreal
Clifford Weil, National Science Foundation
Asela Wijeratne, Arkansas State University
Chathura Wijesinghege, Louisiana State University
Olivia Wilkins, University of Manitoba
Mary Williams, American Society of Plant Biologists
Rafal Woycicki, Applied Omics Wóycicki
Pin-Jou Wu, Max Planck Institute for Developmental Biology
Chi-Chih Wu, Academia Sinica
Kaltra Xhelilaj, Universität Tübingen
Deyu Xie, North Carolina State University
Xiaosa Xu, Cold Spring Harbor Laboratory
Deyang Xu, University of Copenhagen
J Xu, Radboud University
Shuqing Xu, University of Muenster
Xiaocai Xu, Humboldt-Universität zu Berlin
Deyang Xu, N/A
Shouling Xu, Carnegie Institution for Science
Hang Xue, University of California Berkeley
Arpita Yadav, University of Massachusetts Amherst
Lang YANG, University of Hong Kong
Hailong Yang, East Carolina University
Leiyun Yang, Cornell University
Tao Yao, Oak Ridge National Laboratory
Qiuming Yao, University of Nebraska Lincoln
Shohreh Yazdani, Tehran University
Natalie Young, ThermoFisher Scientific
Houlin Yu, University of Massachusetts Amherst
Yanling Yu, University of Freiburg
Hai Ying Yuan, National Research Council Canada
Jay Yung, Carnegie Institution for Science
Argyris Zardilis, Sainsbury Laboratory, University of Cambridge
Junpeng Zhan, Donald Danforth Plant Science Center
Xixi Zhang, Helsinki University
Yunming Zhang, Huazhong Agricultural University
Liechi Zhang, National Institute for Basic Biology
Shiqi Zhang, Boyce Thompson Institute
Wentao Zhang, National Research Council Canada
Guofeng Zhang, University of Freiburg

Zhenzhen Zhang, Carnegie Institution for Science
Zhizhu Zhao, Xiamen University
Yaping Zhou, University of Bonn Crop Functional Genomics
Jie Zhu, University of California Davis
Xiaohong Zhuang, Chinese University of Hong Kong
Nathan Zivi, Swedish University of Agricultural Sciences
Mahdis Zolfaghar, University of Tehran

Appendix S6: PCA Consortium Authors

First Name	M.I.	Last Name	Institution	Country
Pinky		Agarwal	National Institute of Plant Genome Research	India
Amir H.		Ahkami	Pacific Northwest National Laboratory	USA
Jahed		Ahmed	Laboratoire de Biogènese Membranaire, CNRS, Université de Bordeaux	France
Oluwafemi	A.	Alaba	University of Maine	USA
Gazala		Ameen	South Dakota State University	USA
Christopher	R	Anderton	Pacific Northwest National Laboratory	USA
Mario	A	Arteaga-Vazquez	Universidad Veracruzana	México
M. Arif		Ashraf	University of Massachusetts Amherst	USA
Dominique	C	Bergmann	Stanford University	USA
Edoardo		Bertolini	Donald Danforth Plant Science Center	USA
Noel		Blanco-Touriñán	Group of Plant Vascular Development, Swiss Federal Institute of Technology (ETH)	Switzerland
Javier		Brumos	Institute for Plant Molecular and Cell Biology (IBMCP), CSIC	Spain
Alfredo		Cruz-Ramirez	Advanced Genomics Unit CINVESTAV	México
Stefan		de Folter	UGA-Langebio, CINVESTAV	México
Pingtao		Ding	Institute of Biology Leiden, Leiden University	The Netherlands
Camilla		Ferrari	VIB-UGent Center for Plant Systems Biology	Belgium
Stefania		Giacomello	SciLifeLab, KTH Royal Institute of Technology	Sweden
Fabio		Gomez-Cano	Michigan State University	USA
Maria	J	Harrison	Boyce Thompson Institute	USA
Venura		Herath	University of Peradeniya	Sri Lanka
Humberto		Herrera-Ubaldo	UGA-Langebio, CINVESTAV	México
Abhishek		Joshi	Mohanlal Sukhadia University	India
Ramesh		Katam	Florida A&M University	USA

Harmanpreet		Kaur	Punjab agricultural university	India
Mather	A	Khan	Heinrich Heine University	Germany
Dae Kwan		Ko	Michigan State University	USA
Pankaj		Kumar	Dr Yashwant Singh Parmar University of Horticulture and Forestry Nauni Solan Himachal Pradesh	India
Sagar		Kumar	Mata Gujri College, Fatehgarh Sahib, Punjabi University	India
Arun		Kumar	CSIR-Institute of Himalayan Bioresource Technology	India
Dhruv		Lavania	MacEwan University	Canada
Mathew	G	Lewsey	La Trobe University	Australia
Marc		Libault	University of Nebraska-Lincoln	USA
Chien-Yuan		Lin	Lawrence Berkeley National Laboratory	USA
Tie		Liu	University of Florida	USA
Le		Liu	University of Massachusetts Amherst	USA
Ansul		Lokdarshi	Valdosta State University	USA
Ai-My		Luong	Louvain Institute of Biomolecular Sciences, Catholic University of Louvain	Belgium
Sakil		Mahmud	Plant Cell Biology Dept., IZMB, University of Bonn	Germany
Concepcion		Manzano	UC Davis	USA
Devang		Mehta	University of Alberta	Canada
Miguel		Miñambres Martín	Plant Biochemistry Institute HHU University Düsseldorf	Germany
Sushma		Naithani	Oregon State University	USA
Hilde		Nelissen	VIB/Ugent	Belgium
Trevor	M	Nolan	Duke University	USA
Toshihiro		Obata	University of Nebraska-Lincoln	USA
Elsa	Herminia	Quezada-Rodríguez	Universidad Nacional Autónoma de México	México
Atique	ur	Rehman	Bahauddin Zakariya University	Pakistan
Luis	C	Romero	Institute of Plant Biochemistry and Photosynthesis, CSIC-US	Spain

Saroj	K	Sah	Brookhaven National Laboratory	USA
Shyam		Solanki	South Dakota State University	USA
Gary		Stacey	University of Missouri, Columbia	USA
Josh		Strable	North Carolina State University	USA
Tuan	M	Tran	University of South Alabama	USA
Rajiv	Kumar	Tripathi	McGill University	Canada
Klaas		Vandepoele	Department of Plant Biotechnology and Bioinformatics, Ghent University/VIB Center for Plant Systems Biology	Belgium
Tamas		Varga	Pacific Northwest National Laboratory	USA
Marija		Vidovic	Laboratory for Plant Molecular Biology Institute of Molecular Genetics and Genetic Engineering, University of Belgrade	Serbia
Jinzheng		Wang	University of California, Riverside	USA
Olivia		Wilkins	University of Manitoba	Canada
Houlin		Yu	University of Massachusetts Amherst	USA
Hai Ying		Yuan	National Research Council Canada	Canada
Jie		Zhu	University of California, Davis	USA
Ying		Zhu	Pacific Northwest National Laboratory	USA

First Plant Cell Atlas Symposium

Abstract Book

December 9 & 10, 2021

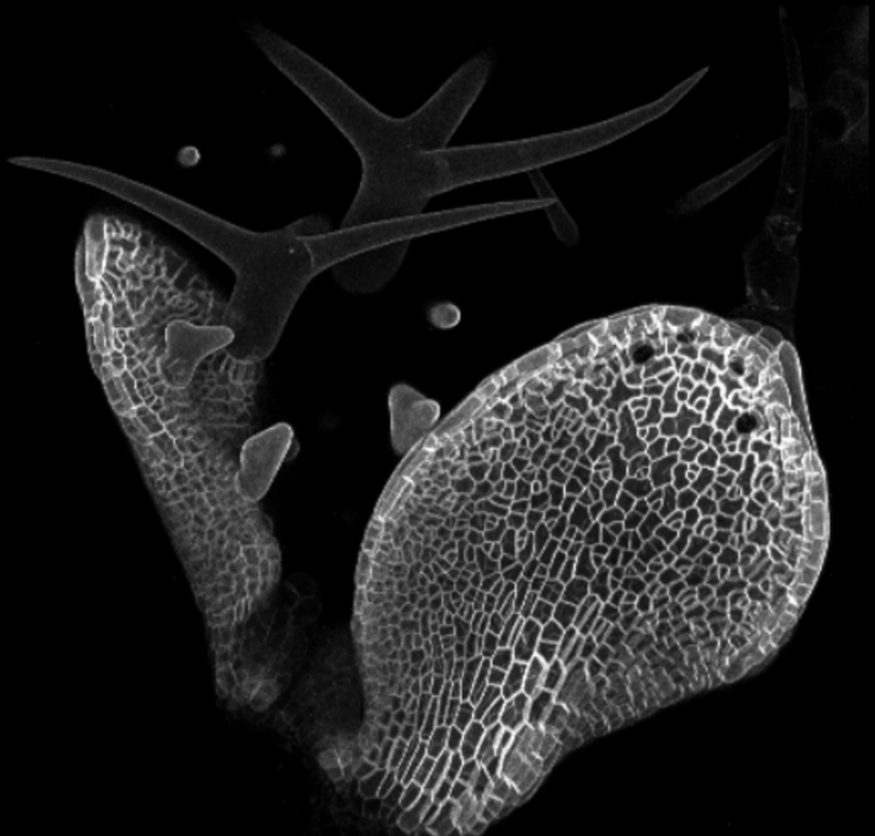

TABLE OF CONTENTS

Dec 9th

**Poster Number** / Abstract Title / Time	
**01**	The role of a conserved HSF:miR169:NF-YA loop during heat stress in *Arabidopsis* and tomato *(Solanum lycopersicum)* (8:30-8:50 AM PT)
**02**	Towards an atlas of REMORIN-nanodomains associated functions (8:30-8:50 AM PT)
**03**	Light-sheet microscopy in plant developmental imaging (8:30-8:50 AM PT)
**04**	Unwinding the heat mediated post-transcriptional regulation of Hsf18 in tomato (8:50-9:10 AM PT)
**05**	Analysis of cell wall remodeling at the plant–rhizobial interface in *Medicago truncatula* (8:50-9:10 AM PT)
**06**	Elucidation of signaling pathways via comparative transcriptome analysis of hypocotyls during the developmental transition of C3 cotyledons to C4 leaves in *Halimocnemis mollissima* (8:50-9:10 AM PT)
**07**	Tale of the nucleus: A nuclear membrane protein is required for correct division plane orientation (9:10-9:30 AM PT)
**08**	Cryo-Electron Microscopy for Structural and Contextual Insights into Plant Cell Biology (9:10-9:30 AM PT)
**09**	*Arabidopsis* CALMODULIN-LIKE 38 Regulates Hypoxia-Induced Autophagy of SUPPRESSOR OF GENE SILENCING 3 Bodies (11:50-12:10 PM PT)
**10**	Interrogating plant hormone and stress responses at cellular resolution using time series single-cell transcriptomics (11:50-12:10 PM PT)
**11**	Application of multiplex fluorescent RNAscope RNA *in situ* hybridization in plants (11:50-12:10 PM PT)
**12**	Identification of new marker genes from plant single-cell RNA-seq data using interpretable machine learning methods (12:10-12:30 PM PT)
**13**	Closing the protein gap in plant chronobiology using time-course BoxCarDIA Proteomics (12:10-12:30 PM PT)

TABLE OF CONTENTS

Dec 10th

**Poster Number** / Abstract Title / Time	
**14**	Unravelling the effect of temperature on flowering regulation at the tissue-specific level (8:30-8:50 AM PT)
**15**	Single-cell resolution of plant response to bacterial infection (8:30-8:50 AM PT)
**16**	An *Arabidopsis* root phloem pole cell atlas reveals *PINEAPPLE* genes as transitioners to autotrophy (8:50-9:10 AM PT)
**17**	Tasselyzer, a machine learning method to quantify anther extrusion in maize, based on PlantCV (8:50-9:10 AM PT)
**18**	Spatial transcriptomics reveals developmental patterns in the tissues of vegetative and reproductive buds of Norway spruce (8:50-9:10 AM PT)
**19**	A single-cell *Arabidopsis* root atlas reveals developmental trajectories in wild type and cell identity mutants (9:10-9:30 AM PT)
**20**	Integrating molecular and mechanical signals in *Arabidopsis* early flower development (9:10-9:30 AM PT)
**21**	Single nuclei transcriptome of the Lesser Duckweed *Lemna minuta* reveals cell trajectories for an entire plant (11:50-12:10 PM PT)
**22**	Regulation of plasmodesmata function at specific cell-cell interfaces in *Arabidopsis* (11:50-12:10 PM PT)
**23**	*In vitro* experiments and kinetic models of pollen hydration mechanics show that MSL8 is not a simple tension-gated osmoregulator (11:50-12:10 PM PT)
**24**	A field-grown rice translatome atlas (12:10-12:30 PM PT)
**25**	The Plant-Fungal Interface: Application of Spatially-Resolved, Single Cell Transcriptomics to the Arbuscular Mycorrhizal Symbiosis (12:10-12:30 PM PT)

APOORVA GUPTA

National Institute of
Plant Genome Research,
India

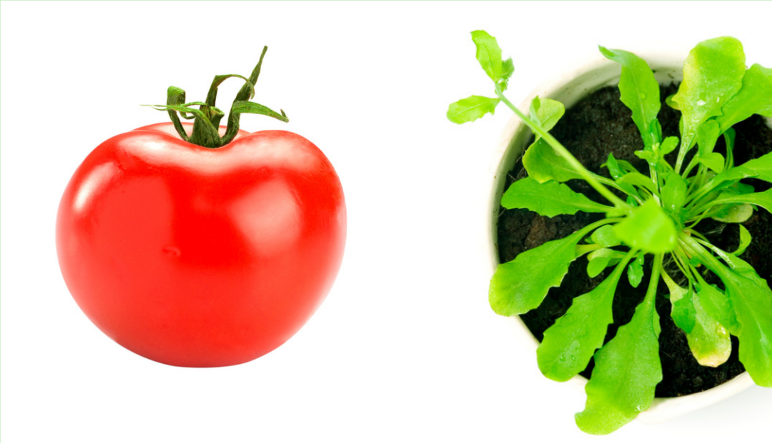

The role of a conserved HSF:miR169:NF-YA loop during heat stress in *Arabidopsis* and tomato (*Solanum lycopersicum*)

Apoorva Gupta[1], Sombir Rao[1], Sarita Jha[1], Chandni Bansal[1], Celine Sorin[2], Martin Crespi[2] and Saloni Mathur[1]
1 National Institute of Plant Genome Research, New Delhi, India
2 Université Paris-Saclay, CNRS, INRAE, Univ Evry, Institute of Plant Sciences Paris-Saclay (IPS2), 91405, Orsay, France

Heat stress adversely affects the productivity of agriculturally important crops worldwide. Regulatory feedbacks are at the basis of different stress and developmental networks in plants. Here, we report that tomato and *Arabidopsis* plants improve their heat stress tolerance through Heat stress transcription factor (HSF)-mediated transcriptional regulation of microRNA169 and post-transcriptional regulation of Nuclear Factor Y-Subunit A (NF-YA) transcription factors. We show that HSFs recognize tomato and *Arabidopsis* MIR169 promoters using yeast one-hybrid/ChIP-qPCR. Silencing tomato HSFs using virus-induced-gene-silencing (VIGS) reduce Sly-MIR169 levels and enhance Sly-NF-YA9/A10 target expression. Further, tomato transgenic plants overexpressing Sly-MIR169 and Sly-NF-YA9/A10-VIGS knock-down tomato plants as well as *Arabidopsis* plants overexpressing At-MIR169d and At-nf-ya2 mutants showed a link with increased heat tolerance. In contrast, *Arabidopsis* plants overexpressing At-NF-YA2, or those expressing a non-cleavable At-NF-YA2 form (miR169- resistant At-NF-YA2) as well as plants inhibited for At-miRNA169d regulation (miR169d mimic plants) were more sensitive to heat stress, highlighting NF-YA as negative regulator of heat tolerance. Furthermore, post-transcriptional cleavage of NF-YA by elevated miR169 levels result in alleviating the repression of heat stress effectors HSFA7a/b in tomato and *Arabidopsis* revealing a retroactive control of HSFs by the miR169:NF-YA node. Hence, a regulatory feedback loop involving HSFs, miR169s and NF-YAs plays a critical role in the regulation of heat stress response in tomato and *Arabidopsis* plants.

KALTRA XHELILAJ

Universität Tübingen, Germany

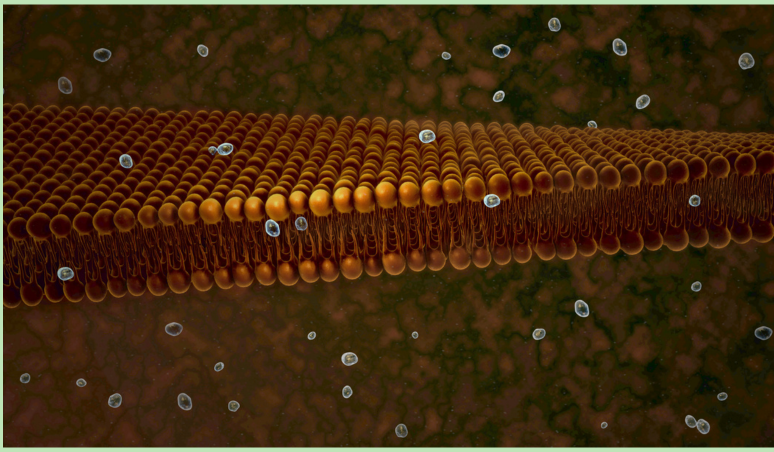

Towards an atlas of REMORIN-nanodomains associated functions

Kaltra Xhelilaj and Julien Gronnier
NanoSignaling Lab, ZMBP, Universität Tübingen, Auf der Morgenstelle 32 72076 Tübingen, Germany

Plasma membrane lipids and proteins are dynamically organized into diverse nano-environment giving rise to fluid molecular patchworks1. These membrane nano-environments are often referred as nanodomains and are characterized by a local composition, lateral organization, and/or dynamics which differ from the average membrane properties1,2. Their formation is regulated as part of developmental programs, and is modulated to respond to stimuli and supports protein function. REMORINs (REMs) constitute a plant specific protein family which recently emerged as regulatory components of immunity, symbiosis, and development3. REMs of different groups tend to form distinct and coexisting NDs which are proposed to host specific signalling pathways4. However, the molecular functions associated with REMs nanodomains largely unknown. To answer this question, we will perform an organism-wide functional characterization of REMs nanodomains architecture, composition, and function. Using live cell imaging approaches and super resolution microscopy, we are characterizing time-resolved and context-depended expression and nanodomain organization of REMs. To identify function associated with distinct REM nanodomains we are coupling single cell transcriptomic and proteomic approaches. Finally, in order to genetically dissect REM NDs associated functions, we are generating a collection of REM mutants using a multiplexed CRISPR strategy. Our project will shed light on REMORIN-mediated regulation of cell surface signaling across different cell types and tissues.

1. Gronnier J, Gerbeau-Pissot P, Germain V, Mongrand S, Simon-Plas F. Divide and Rule: Plant Plasma Membrane Organization. Trends in Plant Science. 2018;23(10):899-917. doi:10.1016/j.tplants.2018.07.007
2. Jaillais Y, Ott T. The nanoscale organization of the plasma membrane and its importance in signaling: A proteolipid perspective. Plant Physiology. 2020;182(4):1682-1696. doi:10.1104/PP.19.01349
3. Gouguet P, Gronnier J, Legrand A, et al. Connecting the dots: From nanodomains to physiological functions of REMORINs. Plant Physiology. 2021;185(3):632-649. doi:10.1093/PLPHYS/KIAA063
4. Jarsch IK, Konrad SSA, Stratil TF, et al. Plasma membranes are Subcompartmentalized into a plethora of coexisting and diverse microdomains in Arabidopsis and Nicotiana benthamiana. Plant Cell. 2014;26(4):1698-1711. doi:10.1105/tpc.114.124446

DR. MIROSLAV OVEČKA

Palacký University Olomouc,
Czech Republic

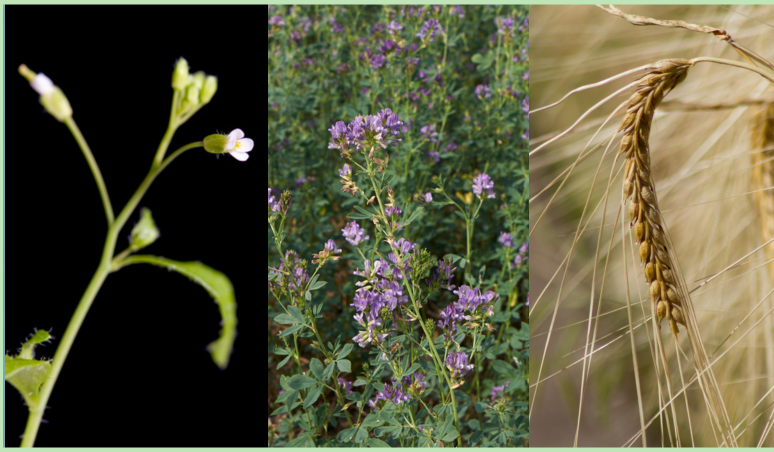

Light-sheet microscopy in plant developmental imaging

Miroslav Ovečka[1], Jiří Sojka[1], Michaela Tichá[1], George Komis[1], Jasim Basheer[1], Cintia Marchetti[2], Olga Šamajová[1], Lenka Kuběnová[1], Jozef Šamaj[1]
1 Department of Cell Biology, Centre of the Region Haná for Biotechnological and Agricultural Research, Faculty of Science, Palacký University Olomouc, Šlechtitelů 27, 783 71 Olomouc, Czech Republic.
2 Centre of the Region Haná for Biotechnological and Agricultural Research, Czech Advanced Technology and Research Institute, Palacký University Olomouc, Šlechtitelů 27, 783 71 Olomouc, Czech Republic

Plant development, a highly dynamic spatio-temporal process, makes live imaging of plants challenging. Light-sheet fluorescence microscopy (LSFM) can visualize plant development at multiscale subcellular, cellular, tissue and organ levels. Here we document the great potential of LSFM in the elucidation of root development including growth of primary roots and formation of root hairs. LSFM is suitable method for developmental imaging of root hairs. Upon stabilization of seedlings with continuous root growth in LSFM chamber, undisturbed development of root hairs and their sustained tip growth is effectively achieved. We show examples of model plant *Arabidopsis thaliana* (L.) Heynh., but also robust crop species *Medicago sativa L.* and *Hordeum vulgare* L. documenting growth and development of crop roots captured by LSFM in real time. We also used LSFM to visualize *M. sativa* interactions with symbiotic nitrogen-fixing bacteria *Sinorhizobium meliloti.* Volumetric imaging of barley root growth and development is shown employing transgenic plants with molecular marker for *in vivo* visualization of actin cytoskeleton. Current trend shifting science from purely basic to applied one is apparent. Advanced imaging of crops is a next milestone in this effort towards improved development, yield, and stress resistance. LSFM is a new promising tool to reveal plant complexity by long-term live volumetric imaging of intact developing plants.

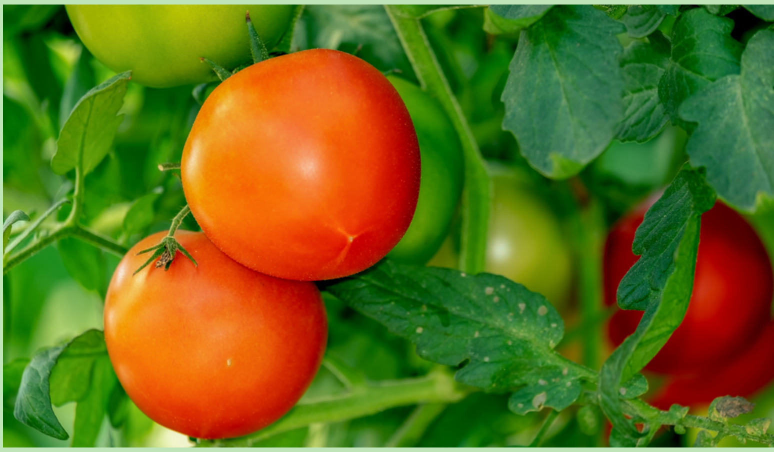

JAISHRI RUBINA DAS

National Institute of
Plant Genome Research,
India

Unwinding the heat mediated post-transcriptional regulation of Hsf18 in tomato

Jaishri Rubina Das, Sombir Rao and Saloni Mathur
National Institute of Plant Genome Research, New Delhi, India

Heat stress (HS) poses a major threat to the production of important horticultural crops like tomato. To combat stress conditions, plant cell undergo extensive reprogramming of molecular events. One such adaptive strategy is post-transcriptional regulation by alternative-splicing (AS), which can tremendously increase the potential of an organism's transcriptome as well as proteome. Experimental validation of heat stress transcription factor (Hsf) AS-forms in tomato highlighted that some AS-variants express differentially under different stress conditions and different tissues. In-depth analysis of Hsf18 (a repressor) identified five splice variants (Hsf18-V3, Hsf18-V6, Hsf18-V7, Hsf18-V17 and Hsf18-V23). All of these variants showed higher expression under HS, contrary to the Hsf18-FL.

Moreover, Hsf18-FL is targeted by one microRNA (miR-sm-1) while the variants lack the target site of miRNA and thus bypass the targeting by miRNA. The variants Hsf18- V3/V6/V7 have coding potential, DBD (DNA binding domain) and NLS (nuclear localization signal) like the full-length Hsf18 and are localized in nucleus. The alternative splicing of Hsf18 was validated *in-planta* by Western blotting using the Hsf18 genomic context:GFP construct and GFP antibody. Interestingly, we also found that the complementary strand of Hsf18 locus transcribe lncRNA, which is also HS responsive. Promoter-Hsf18 (Pro-Hsf18) is non HS-responsive but promoter-lncRNA (Pro-lncRNA) was found to be responsive under HS. Yeast-one hybrid assay confirmed the binding of Hsf18-FL and Hsf18-V3/V6/V7 on Pro-lncRNA. Moreover, the *in-planta* assay highlighted that the lncRNA is regulated negatively by Hsf18-FL which correlates with the fact that Hsf18-FL is a repressor whereas positively by the variants Hsf18-V3/V6/V7 which lack the repressor domain and thus the variants can be considered as activators. The functional characterization of Hsf18-FL, Hsf18- V7 and lncRNA overexpression lines confirmed that Hsf18-FL seedlings showed thermosensitivity whereas, overexpression of Hsf18-V7 and lncRNA provided HS tolerance to the plants. Thus, we concluded that Hsf18-FL is a negative regulator of HS while Hsf18- V7 and lncRNA are positive regulator of HS.

DR. CHAO SU

University of Freiburg, Germany

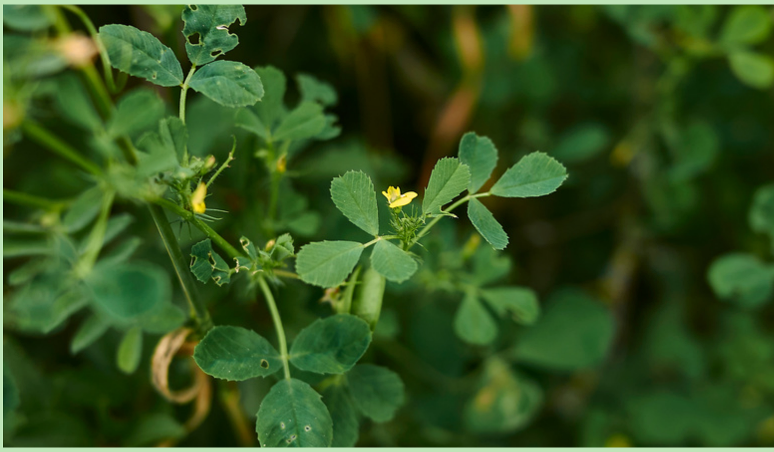

Analysis of cell wall remodeling at the plant-rhizobial interface in *Medicago truncatula*

Chao Su[1], Jenny Wietschorke[1], Marta Rodriguez-Franco[1], Guofeng Zhang[1], Wei Yang[2] , Pengbo Liang[1], Leonard Uhler[1], Xia Li[2], Thomas Ott[1,3]
1 Cell Biology, Faculty of Biology, University of Freiburg, 79104 Freiburg, Germany
2 State Key Laboratory of Agricultural Microbiology, College of Plant Science and Technology, Huazhong Agricultural University, No. 1 Shizishan Road, Hongshan District, Wuhan, Hubei 430070, P.R. China
3 CIBSS – Centre of Integrative Biological Signalling Studies, University of Freiburg, 79104 Freiburg, Germany

Intracellular colonization of plant cells symbiotic bacteria like rhizobia requires local cell wall modification to allow transcellular passage. Here, we investigated the local cell wall modification from the cellular level by cell biology approaches. We found that un-esterified pectin was highly accumulated around the infection structures including the infection threads (ITs) and IT-host cell conjunction sites. Further, immunofluorescence labeling with antibodies LM19 and 2F4 indicates that the unesterified pectin concentrated at the penetration sites was associated with Ca2+, thereby increasing the stiffness of the cell wall. In addition, we identified a symbiotic pectin methylesterase (SyPME) which potentially can cooperate with nodule pectate lyase (NPL) to medicate the local cell wall pectin modification at the plant-rhizobial interface.

MAHDIS ZOLFAGHAR

University of Tehran,
Iran

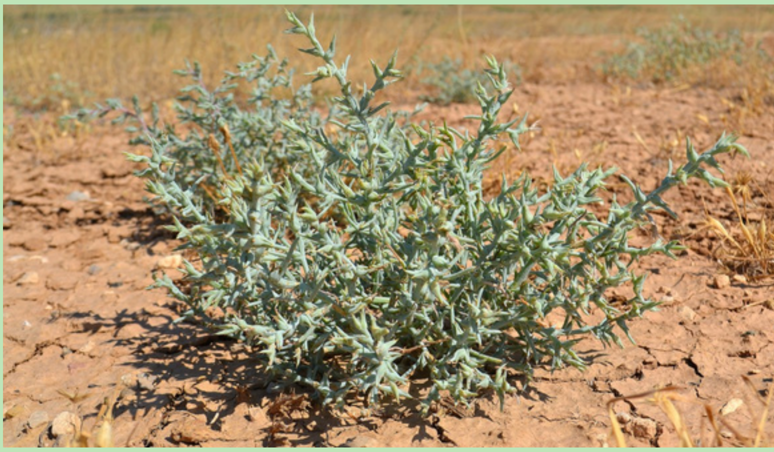

Elucidation of signaling pathways via comparative transcriptome analysis of hypocotyls during the developmental transition of C3 cotyledons to C4 leaves in *Halimocnemis mollissima*

Mahdis Zolfaghar[1], Mohammad Reza Ghaffari[2] , Ali Mohammad Banaei-Moghaddam[1]
1 Institute of Biochemistry and Biophysics, Laboratory of Genomics and Epigenomics (LGE), University of Tehran, Tehran, Iran
2 Department of Systems Biology, Agricultural Biotechnology Research Institute of Iran (ABRII), Agricultural Research, Karaj, Iran

C4 photosynthesis is a syndrome that increases CO_2 fixation efficiency by spatial separation of photosynthesis phases into the mesophyll and bundle-sheath cells. Besides, C4 plants are more efficient in water and nitrogen consumption. The C4 characteristics have evolved from C3 progenitors in more than 60 independent lineages. Therefore they are the most striking instances of convergent evolution. The unique structure of Kranz anatomy in C4 leaves
facilitates the C4 biochemical features. Nevertheless, exploring the evolutionary paths and identifying the global regulators of key features of C4 photosynthesis is constrained by the phylogenetic noises in comparative studies. In its life cycle, *H. mollissima* performs both C3 (in cotyledons) and C4 (in first leaves) photosynthesis. Such species provided an excellent model to study the regulatory networks during C3 to C4 transition. Accordingly, we aimed to investigate probable long-distance communication pathways involved in this transition through transcriptome analysis of hypocotyl. Thus, we extracted RNA from hypocotyls before and after the first leaves' formation. High-quality RNA was sequenced by Illumina Hi-seq 2000 sequencer.

We identified differentially expressed genes that belong to various regulatory and signaling pathways. Two members of the GRAS family of transcription factors, SHORT-ROOT (SHR) and SCARECROW (SCR), were more expressed in hypocotyls after formation of first leaves. It has already been shown that SHR is a mobile transcription factor and SCR controls Kranz anatomy in maize leaves. Some of the genes involved in the biosynthesis and transportation of phytohormones like ethylene, strigolactone, and jasmonic acid were also up-regulated in hypocotyl after C4 leaves formation. Transcripts of signal peptides, including several cysteine rich peptides, had a higher abundance in hypocotyls before the appearance of C4 leaves. The functional annotation of the differentially expressed transcripts implies the existence of a root to-shoot-to-root circuit and a possible role of mobile macromolecules in the regulation of this developmental transition in the photosynthesis strategy. Further investigations are required to elucidate the exact role of identified candidates in the manifestation of C4-associated anatomical and biochemical features.

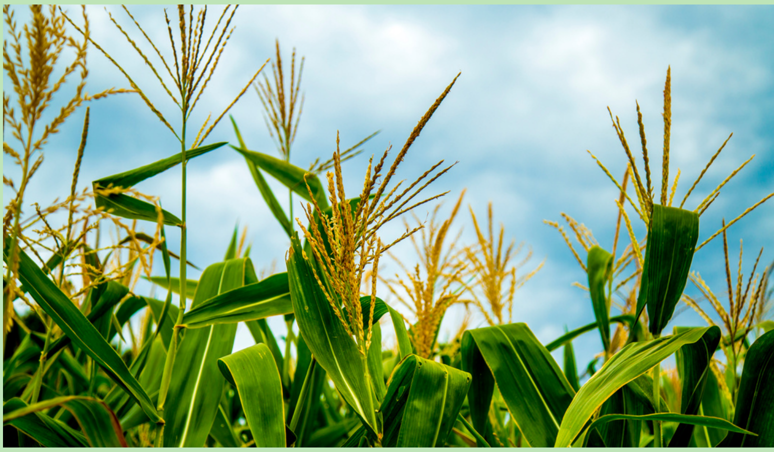

DR. ARIF ASHRAF

University of Massachusetts Amherst, USA

Tale of the nucleus: A nuclear membrane protein is required for correct division plane orientation

M. Arif Ashraf, Michelle Facette
Department of Biology, University of Massachusetts Amherst, Amherst, MA, USA

Both plants and animals rely on asymmetric cell division to generate new cell types, which is a core characteristic of multicellular organisms. Prior to asymmetric cell division, cell polarity is established. Cell polarity establishment and asymmetric cell division are universally important, although proteins important for polarity differ in plants and animals. *Zea mays* stomatal development serves as an excellent plant model system for asymmetric cell division. In this process, the nucleus migrates to the future division site after polarity establishment and before cytokinesis. In this study, we examined a mutant of the outer nuclear membrane protein, which is part of the LINC (linker of nucleuoskeleton and cytoskeleton) complex, which localizes to the nuclear envelope in interphase cells. Previously, plants harboring mutations in m*lks2* (Maize LINC KASH AtSINE-like) were observed to have abnormal stomata (Gumber et al. 2019 Nucleus). We confirmed stomatal defects such as abnormal subsidiary cell size and shape, aborted guard mother cell, and extra inter-stomatal cells. We used cell markers to pinpoint the precise defects that lead to abnormal asymmetric divisions. Early markers of cell polarization, including actin patch formation, are normal in *mlks2* mutants. Nuclear polarization is impaired. Notably, our data indicate that wrong preprophase band (ppb) forms in the cells with abnormal nuclear positioning (offset and completely unpolarized). Additionally, spindle orientation is more variable in *mlks2* mutants. Future studies will use time-lapse imaging to quantify defects in individual cell division events (ppb, spindle, phragmoplast, cytokinesis). Since we observe division defects after nuclear envelope breakdown, localization of MLKS2 and other LINC complex members during plant cell division is relevant. We will determine cellular localization of ZmMLKS2 using immunostaining and tobacco BY2 (bright yellow 2) cell during mitosis. Altogether, our study will help to dissect the role of nuclear movements during different steps of asymmetric cell division including polarization, division plane establishment, division plane maintenance, mitosis, and cytokinesis.

DR. NATALIE YOUNG

ThermoFisher Scientific, USA

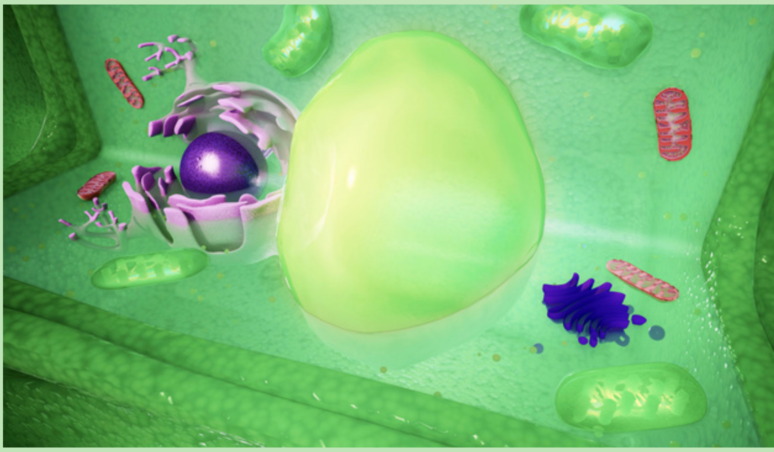

Cryo-Electron Microscopy for Structural and Contextual Insights into Plant Cell Biology

Natalie Young, PhD
ThermoFisher Scientific

The Plant Cell Atlas project's goal is to advance a more comprehensive molecular understanding of plant cellular structure and function. Although there are many tools for studying plant cellular structure and function, electron microscopy (EM) is proven as a fundamental technique for obtaining high resolution structural information. More specifically, cryogenic-EM (cryo-EM) allows for the determination of plant cell structure under native conditions. Particularly significant is cryo-EM's ability to observe a wide range of detail, from the structural details of proteins (with single particle analysis) to their cellular context (with tomography), all the way up to the overall structure of the plant (large volume analysis), providing a full understanding of fundamental interactions and mechanisms. In the fields of plant biology and crop science, these insights can shed light not just on cellular function but also interaction of unknown chemical substances of pathogens with the plant. For example, electron microscopy can reveal the interaction between viral/fungal species and the crop, informing breakthroughs in disease prevention that result in increased crop and agricultural product yield. Here, I show how the cryo-EM workflow, for both single particle analysis and tomography, can be applied to understand plant cell structure and function.

DR. STERLING FIELD

Carnegie Institution for Science, USA

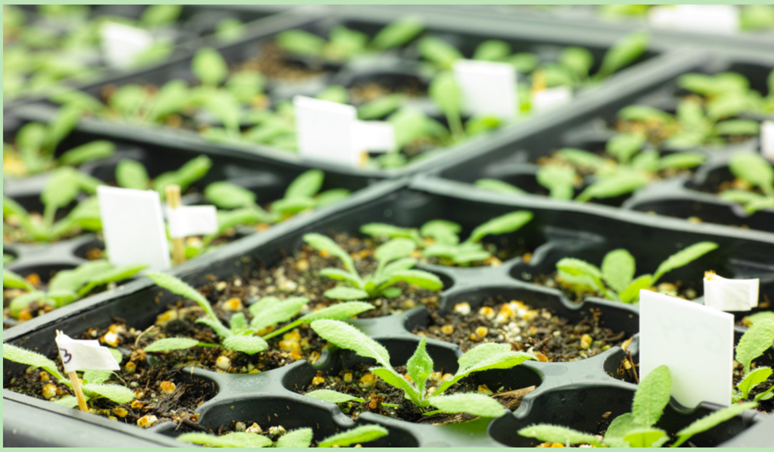

Arabidopsis CALMODULIN-LIKE 38 Regulates Hypoxia-Induced Autophagy of SUPPRESSOR OF GENE SILENCING 3 Bodies

S. Field, W. C. Conner, D. M. Roberts.

During the energy crisis associated with submergence stress, plants restrict mRNA translation and rapidly accumulate stress granules that act as storage hubs for arrested mRNA complexes. One of the proteins associated with hypoxia-induced stress granules in *Arabidopsis thaliana* is the calcium-sensor protein CALMODULIN-LIKE 38 (CML38). Here, we show that SUPPRESSOR OF GENE SILENCING 3 (SGS3) is a CML38-binding protein, and that SGS3 and CML38 co-localize within hypoxia-induced RNA stress granule-like structures. Hypoxia-induced SGS3 granules are subject to turnover by autophagy, and this requires both CML38 as well as the AAA+-ATPase CELL DIVISION CYCLE 48A (CDC48A). CML38 also interacts directly with CDC48A, and CML38 recruits CDC48A to CML38 granules in planta. Together, this work demonstrates that SGS3 associates with stress granule-like structures during hypoxia stress that are subject to degradation by CML38 and CDC48-dependent autophagy. Further, the work identifies direct regulatory targets for the hypoxia calcium-sensor CML38, and suggest that CML38 association with stress granules and associated regulation of autophagy may be part of the RNA regulatory program during hypoxia stress.

DR. TREVOR NOLAN

Duke University,
USA

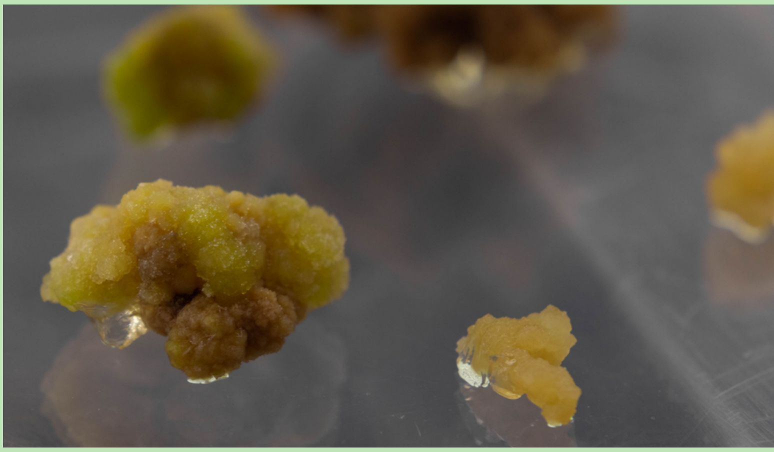

Interrogating plant hormone and stress responses at cellular resolution using time series single-cell transcriptomics

S.Trevor M Nolan[1], Nemanja Vukašinović[2], Che-Wei Hsu[3], Isaiah W Taylor[1], Rachel Shahan[1], Laura Greenstreet [4], Anton Afanassiev[4], Geoffrey Schiebinger[4], Uwe Ohler[3], Eugenia Russinova[2], Philip N Benfey[1]
1 Duke University and Howard Hughes Medical Institute, Department of Biology, Durham, NC,
2 Department of Plant Biotechnology and Bioinformatics, Ghent University, Ghent, Belgium,
3 Humboldt Universität zu Berlin, Department of Biology, Berlin, Germany,
4 University of British Columbia, Department of Mathematics, Vancouver, Canada

A fundamental question in developmental biology is how the progeny of stem cells become differentiated tissues. The *Arabidopsis* root is a tractable model to address this question due to its simple organization and defined cell lineages. We recently constructed an integrated atlas of more than 110,000 cells using single-cell RNA-seq, which highlighted the continuous nature of development in the *Arabidopsis* root and provided new insights into cell identity acquisition (Shahan et al., 2020, https://doi.org/10.1101/2020.06.29.178863). To investigate how cell identity and hormone signaling affect one another, we tested responses to Brassinosteroids (BRs), a group of plant steroid hormones. BRs regulate diverse processes such as cell division, cell elongation, and differentiation by controlling the activities of BES1 and BZR1 family transcription factors, which in turn mediate the expression of thousands of genes. The gene regulatory networks that control the diverse processes regulated by BRs are only partially understood, but likely involve additional context-specific regulatory factors. To further define spatiotemporal BR responses, we performed a time series single-cell RNA-seq experiment following BR treatment. Annotation enabled by our high-quality reference atlas allowed us to query BR regulated gene expression among the majority of cell types and developmental stages of the root, revealing both known and novel hotspots of BR signaling. We then used gene regulatory network inference approaches to prioritize cell-type-specific transcription factors. Subsequent experimental characterization revealed a role for two transcription factors in BR-mediated cell elongation. We also performed a single-cell RNA-seq time course following osmotic stress treatments to mimic drought, providing insight into how cell identity and environmental responses are intertwined. Candidate regulators from these datasets represent an opportunity to engineer cell-type-specific stress responses. When combined, these datasets represent more than 300,000 single-cell transcriptomes, providing a view of hormone and stress-mediated gene expression at unprecedented resolution.

DR. SHYAM SOLANKI

South Dakota State University, USA

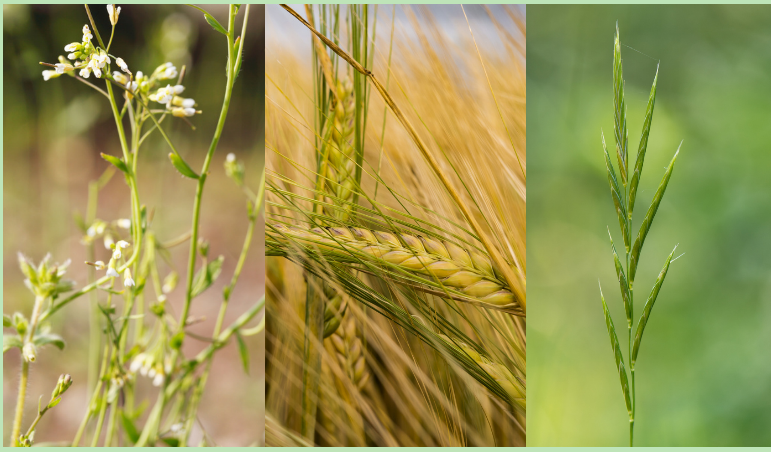

Application of multiplex fluorescent RNAscope RNA *in situ* hybridization in plants

Shyam Solanki[1], Gazala Ameen[1], Pawel Borowicz[2], Karen Sanguinet[3] and Robert S. Brueggeman[3]
1 Department of Agronomy, Horticulture and Plant Science, South Dakota State University, Brookings, SD 57007, USA
2 Department of Animal Sciences, North Dakota State University, Fargo, ND 58108-6050, USA
3 Department of Crop and Soil Science, Washington state University, Pullman, WA 99163, USA

Spatio-temporal expression profiling of RNA and proteins is a key to determining the site-specific function of these biomolecules. The RNA *in-situ* location-to-function paradigm is challenging to explore in the plant tissues, and often lacks sensitivity and reliable protocol reproducibility. The RNAscope RNA *in situ* hybridization (ISH) assay is capable of specifically visualizing multiple RNA species in a tissue or cell type and utilizes RNAscope 'Z' oligonucleotide RNA probes consisting of ~ 18–25 bases complementary to the target sequence. This probe bases assay is capable of highly sensitive target detection based on RNA species-specific fluorescence amplification and visualization. We explored RNAscope assay in plants to determine the site-specific mRNA expression of various candidate genes in *Arabidopsis*, barley, and brachypodium tissue types. We optimized the assay protocol on the leaf and root tissues and showed its suitability to capture site-specific expression of either very low or highly expressed genes. Currently, we are employing the simultaneous RNA-protein co detection assay in stomatal and epidermal cells of wheat and barley to validate the spatiotemporal function of key genes associated with target plant species. Complementation of single-cell omics technologies with bio-molecule visualization techniques such as simultaneous multiplex RNAscope RNA-protein co-detection assay would provide an excellent combination for high throughput validation of gene function in specific plant cells, a key goal of the plant cell atlas initiative.

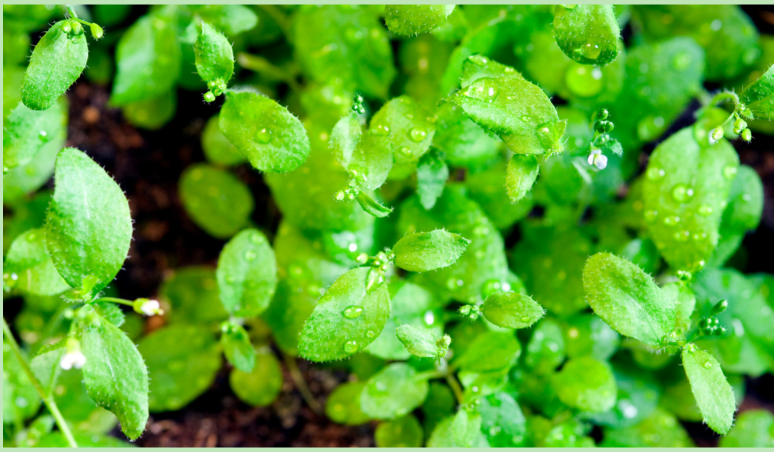

DR. SONG LI

Virginia Tech,
USA

Identification of new marker genes from plant single-cell RNA-seq data using interpretable machine learning methods

Haidong Yan[1], Jiyoung Lee[1,2], Qi Song [1,2], Qi Li[1], John Schiefelbein [3], Bingyu Zhao[1], Song Li [1,2]
1 School of Plant and Environmental Sciences (SPES).
2 Graduate program in Genetics, Bioinformatics and Computational Biology (GBCB),
3 Department of Molecular, Cellular, and Developmental Biology, University of Michigan. Ann Arbor, MI 48109.

An essential step in the analysis of single-cell RNA sequencing data is to classify specific cell types with marker genes. In this study, we have developed a machine learning pipeline called Single cell Predictive markers (SPmarker) to identify novel cell-type marker genes in the *Arabidopsis* root. Unlike traditional approaches, our method uses interpretable machine learning methods to select marker genes. We have demonstrated that our method can (1) assign cell types based on cells that were labeled using published methods, (2) project cell types identified by trajectory analysis from one dataset to other datasets, and (3) assign cell types based on internal GFP markers. Using SPmarker, we have identified hundreds of new marker genes that were not identified before. As compared to known marker genes, we have found more orthologous genes of these new marker genes in corresponding rice single cell clusters. We have also found 172 new marker genes for trichoblast in five non-*Arabidopsis* species, which expands the number of high quality marker genes for this cell type by 35-154%. Our results represent a new approach to identify cell-type marker genes from scRNA-seq data to facilitate cross-species mapping of scRNA-seq data in plants. The computational pipeline is freely available at this github repository: https://github.com/LiLabAtVT/SPMarker

DR. DEVANG MEHTA

University of Alberta, Canada

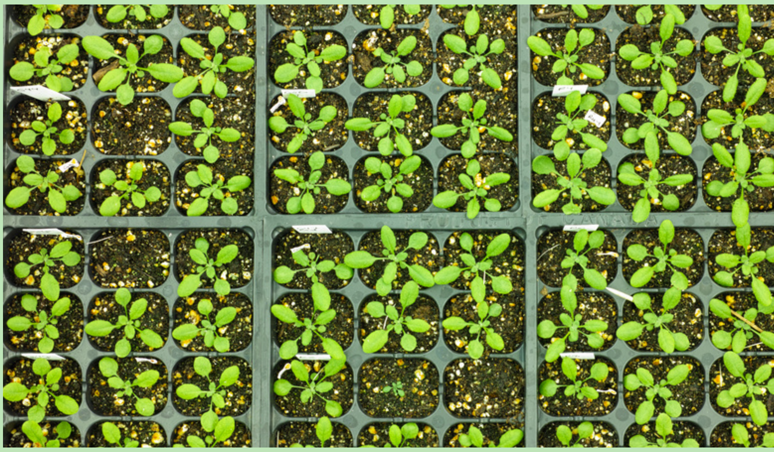

Closing the protein gap in plant chronobiology using time-course BoxCarDIA Proteomics

Devang Mehta, Maria Rodriguez, Sabine Scandola, R. Glen Uhrig
University of Alberta, Department of Biological Sciences, Edmonton, Canada

Over the last decade transcriptomics technologies have powered a revolution in our understanding of the plant circadian clock and chronobiology in model plants and crop systems. This includes a clearer picture of anticipatory (circadian) and reactive (diel) changes in plant gene expression as well as the role of core clock transcription factors. However, transcript-level data is inadequate to provide a complete description of circadian regulation and can mask protein-level events that directly impact plant physiology. Indeed, initial proteomics data from wild-type *Arabidopsis* plants have found an intriguing lack of protein-level oscillation at the same scale as transcriptional oscillation. Phosphoproteomics studies have also found large diel changes in global protein phosphorylation suggesting that circadian post-transcriptional and post-translational mechanisms (PTMs) play a key role in regulating plant circadian outcomes. Here we present data from a large-scale study profiling the diel proteomes of wild-type and circadian clock mutants of *Arabidopsis* for the first time. To execute such complex multivariate time-course experimentation we also developed a new label-free proteomics workflow called BoxCarDIA that enables us to achieve complete protein profiles without the use of data imputation. Finally, our results provide a first look at the impacts of the core clock components at the protein-level revealing new insights into disconnects between the circadian transcriptome and proteome. Our results argue for greater focus on protein and PTM-level circadian dynamics in order to close the protein gap in plant chronobiology.

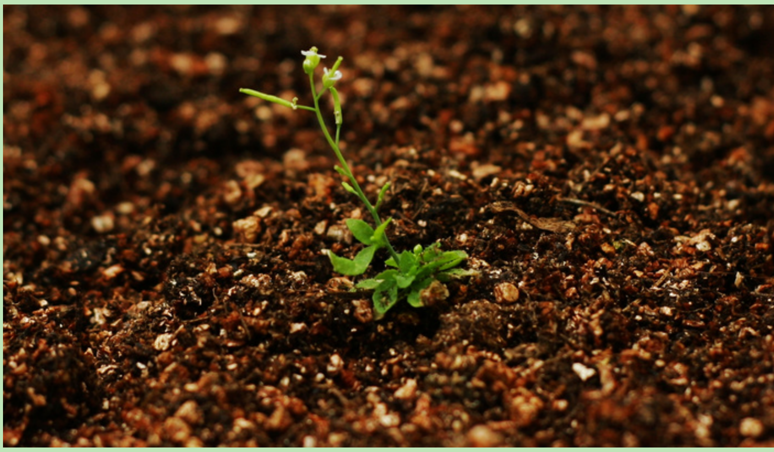

DR. IRENE MARTINEZ-FERNANDEZ

Umeå University,
Sweden

Unravelling the effect of temperature on flowering regulation at the tissue-specific level

Irene Martinez-Fernandez, Ruben Benstein, Joanne Lee, Markus Schmid
Umeå Plant Science Centre.

Development of plants progresses in distinct phases, and transition between these phases is usually irreversible and therefore tightly regulated.Over the years, numerous genes involved in the induction of flowering have been identified in plant model species such as *Arabidopsis thaliana*. Initially these genes were assigned to genetically distinct pathways responding to specific environmental signals, such as the day length (photoperiod) and prolonged periods of cold (vernalization), or to endogenous signals including hormones and other metabolites. More recently it has now become clear that these pathways do not function independently of each other, but rather form a complex regulatory network that eventually controls the activity of what is now referred to as integrator genes. However, most research so far has been performed at the whole-plant level or at best in complex tissues, which can be misleading given that the decision to initiate flowering is made in selected tissues, in particular the phloem companion cells in the leaf vasculature and the shoot apical meristem. For that reason, studies at a tissue-specific level are required. We are developing a system-wide approach to unravel the complex molecular mechanisms that govern the regulation of flowering at the tissue-specific level. Using reporter lines and fluorescent-activated cell sorting (FACS) we are isolating cells from the shoot apical meristem to perform transcriptome analyses and investigate changes in gene expression in response to temperature variation during the transition to flowering.

DR. JIE ZHU

University of California, Davis, USA

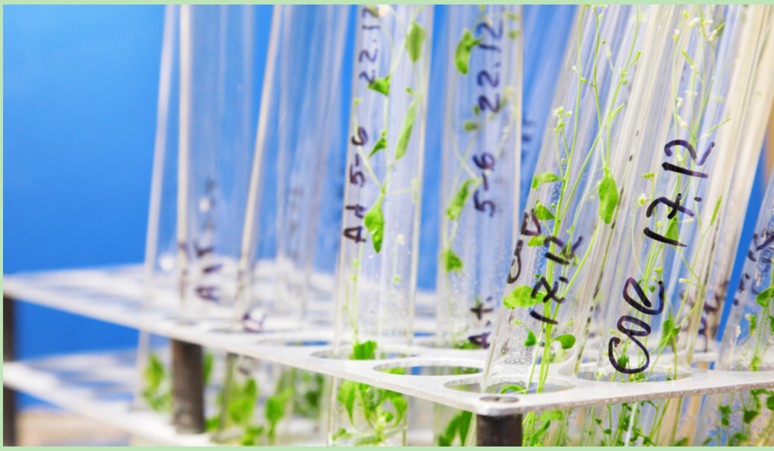

Single-cell resolution of plant response to bacterial infection

Jie Zhu[1], Signe Lolle[1], Benjamin Cole[2], Gitta Coaker[1]
1 Department of Plant Pathology, University of California, One Shields Avenue, Davis, CA 95616, USA
2 DOE Joint Genome Institute, Berkeley, CA 94720, USA

A plant's response to pathogen infection varies by cell and tissue type, yet heterogeneity in pathogen response among cells is not well resolved. Here, we exposed *Arabidopsis* leaf tissue to the bacterial pathogen *Pseudomonas syringae* pv. tomato DC3000 (or a mock treatment) at an early infection stage (24 hours), and profiled the transcriptomes of over 11,000 individual cells using single-cell RNA sequencing, specifically targeting mesophyll cells. We identified most major leaf cell types, with a large proportion of the cell population corresponding to mesophyll identity. Integrative analysis of cell populations from *Pst* DC3000- and mock-inoculated leaves revealed several distinct clusters highly enriched for cells from inoculated leaves, indicating a major shift in mesophyll cells' transcriptomes upon pathogen exposure. Among these, we identified three clusters of cells whose transcriptomic signatures were consistent with an immune response, indicating that *Arabidopsis* perceives and attempts to mount a defense response to virulent *Pst* DC3000. A separate set of cells (the largest pathogen-associated cell cluster) is enriched for transcripts manipulated by *Pst* DC3000 to promote disease including biological processes related to water transport and pathogenesis. Taken together, these data suggest a heterogeneous mixture of immune responsive and susceptible cells within the same tissue. We investigated the expression of *FRK1,* which was expressed in immune cell clusters and is a defense marker gene. Using confocal imaging of the *Pst* DC3000-mCherry inoculated transgenic *Arabidopsis* expressing the *FRK1* fluorescent reporter, we determined that *FRK1* was expressed in surrounding cells of the substomatal cavity colonized by bacteria, suggesting that plant cells within immunity clusters are likely to be sites of early pathogen invasion. Overall, our work uncovers cellular heterogeneity within an infected leaf and provides a unique insight into plant differential responses to infection.

DR. SOFIA OTERO

University of Cambridge - SLCU, UK

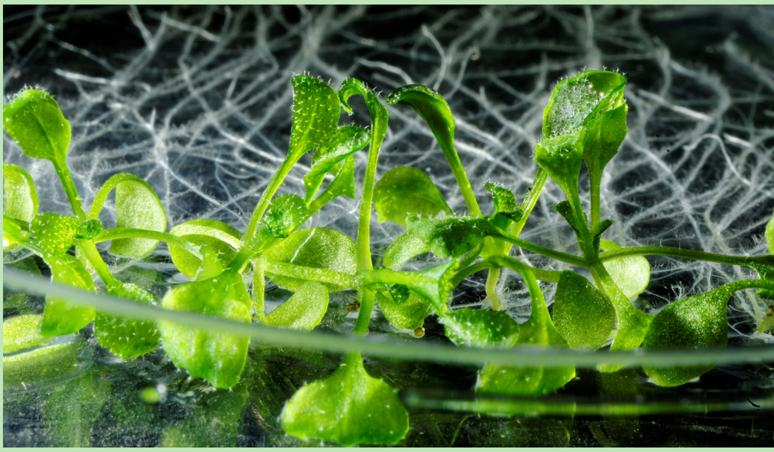

An *Arabidopsis* root phloem pole cell atlas reveals *PINEAPPLE* genes as transitioners to autotrophy

Sofia Otero[1], Iris Sevilem[2,3], Pawel Roszak[1], Yipeng Lu[1], Valerio Di Vittori[4,5], Matthieu Bourdon[1], Lothar Kalmbach[1], Bernhard Blob[1], Jung-ok Heo[1], Federico Peruzzo[6], Thomas Laux[6], Alisdair R. Fernie[4], Hugo Tavares[1,7], Yka Helariutta [1,2]
1 The Sainsbury Laboratory, University of Cambridge, Cambridge, United Kingdom.
2 Institute of Biotechnology, HiLIFE/Organismal and Evolutionary Biology Research Programme, Faculty of Biological and Environmental Sciences, Viikki Plant Science Centre, University of Helsinki, Helsinki, Finland.
3 Present address: Department of Plant Biology and Genome Center, University of California, Davis, California 95616
4 Max-Planck-Institute of Molecular Plant Physiology, Am Müehlenberg 1, Potsdam-Golm, 14476 Germany.
5 Dipartimento di Scienze Agrarie, Alimentari ed Ambientali, Università Politecnica delle Marche, Ancona, Italy
6 BIOSS Centre for Biological Signalling Studies, Faculty of Biology, Albert-Ludwigs-University Freiburg, Schanzlestrasse 1, 7 9104 Freiburg, Germany.
7 Present address: Department of Genetics, University of Cambridge, Cambridge, United Kingdom.

Single cell sequencing has recently allowed the generation of exhaustive root cell atlases. However, some cell types are elusive and remain underrepresented. Here, we use a second generation single cell approach, where we zoom in on the root transcriptome sorting with specific markers to profile the phloem poles at an unprecedented resolution. Our data highlight the similarities among the developmental trajectories and gene regulatory networks communal to protophloem sieve element (PSE) adjacent lineages in relation to PSE enucleation, a key event in phloem biology. As a signature for early PSE-adjacent lineages, we have identified a set of DNA-binding with one finger (DOF) transcription factors, the PINEAPPLEs (PAPL), that act downstream of *PHLOEM EARLY DOF* (PEAR) genes, and are important to guarantee a proper root nutrition in the transition to autotrophy.

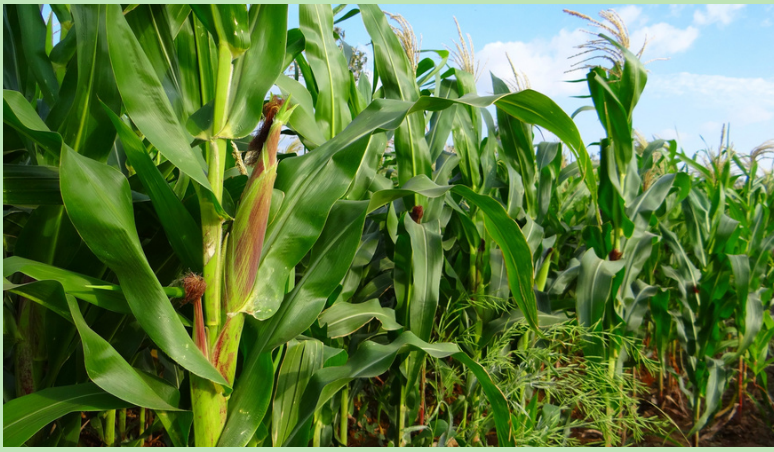

DR. CHONG TENG

Donald Danforth Plant Science Center, USA

Tasselyzer, a machine learning method to quantify anther extrusion in maize, based on PlantCV

Chong Teng[1], Noah Fahlgren[1], Blake C. Meyers[1,2]
1 Donald Danforth Plant Science Center, 975 N. Warson Rd, St. Louis, MO 63132, USA
2 University of Missouri – Columbia, Division of Plant Sciences, 52 Agriculture Lab, Columbia, MO 65211, USA.

Male fertility in maize is controlled by development and genetic programming and is directly impacted by environmental factors such as light, temperature, water, and nutrient availability; the control of this trait has substantial agronomic utility. Maize anthers emerge from male florets, which are clustered to form the tassel at the top of the plant separated from the female ear. Quantification of anther extrusion is one important aspect in the determination of male fertility. To address the lack of an automated method to measure anther extrusion on a large scale, we developed 'Tasselyzer', a quantitative, image-based color trait analysis pipeline for tassel image segmentation, based on the existing PlantCV platform, and we applied it to determine the proportion of anther extrusion. We evaluated Tasselyzer in maize during the seven-day period of pollen shedding as well as in the temperature-sensitive male sterile mutant *dcl5.* With tassel images obtained with a smart phone camera, we show that the anther scores positively correlate with anther extrusion, and such methods can be used to measure environmental impacts on the dcl5 mutant. Altogether, this work establishes an automated and inexpensive method to quantify anther extrusion in maize, which would be useful for research and breeding.

NATHAN ZIVI

Swedish University of Agricultural Sciences, Sweden

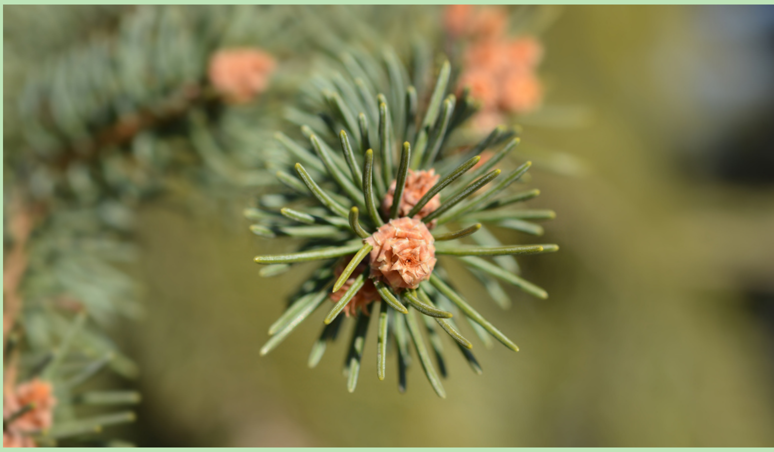

Spatial transcriptomics reveals developmental patterns in the tissues of vegetative and reproductive buds of Norway spruce

Nathan Zivi[1], Yuvarani Masarapu[2] , Sami Saarenpää[2] , Alina Orozco[2] , Jens Sundström[1], Stefania Giacomello[2]
1Swedish University of Agricultural Sciences, Department of Plant Biology, PO Box 7080, SE-750 07 Uppsala, Sweden
2 KTH Royal Institute of Technology, School of Engineering Sciences in Chemistry, Biotechnology and Health (CBH), Department of Gene Technology, Tomtebodavägen 23, SE-171 65 Solna, Sweden

Vegetative and reproductive development in Norway spruce (*Picea abies*) is difficult to study. Trees are tall, have long juvenile periods, and produce female cones infrequently. Additionally, there is a lack of genetic studies and its massive, 20Gbp genome has not been thoroughly annotated. To address these problems, spatial transcriptomics was performed on developing buds (representing female cones, vegetative growth, and a naturally occurring, early female cone producing mutant) at 3 time points in the fall. The buds ranged in size from barely meristems to fully formed buds with visually recognizable tissues and organs easily seen under the microscope after taking longitudinal sections. When the transcriptome from these sections is clustered, the tissue patterns seen visually also appear in the clusters. Of the 16 clusters, 5 represent the pith, 5 represent vascular tissues, 2 represent lateral organs (needles, bracts, and ovuliferous scales), and 2 represent epidermal/meristem tissues. The final 2 clusters did not fall into a specific tissue type. The top genes in each cluster tend to follow genes characteristic of the tissue type the cluster represents. For example, the clusters in vascular tissues have upregulated genes involved in the transport of sugars, hormones, especially auxin, and water (ex. aquaporins). Examination of the clusters that overlap with female organs revealed genes related to female development. Some of these female development genes, like the transcription factor DAL2, show a temporal expression pattern as well as a spatial one. In order to better visualize expression patterns, a shiny app was developed that allows the user to see where in each tissue and time point a particular gene is expressed. Using this tool, DAL2 can be seen to express lowly in August, but extremely highly in October, and almost exclusively in the female organs of female cones. From a broad look at gene expression in different tissue types to the exact expression of an individual gene, spatial transcriptomics allows for a thorough examination of developmental patterns in a previously difficult to study plant with high precision and throughput.

DR. RACHEL SHAHAN

Duke University,
USA

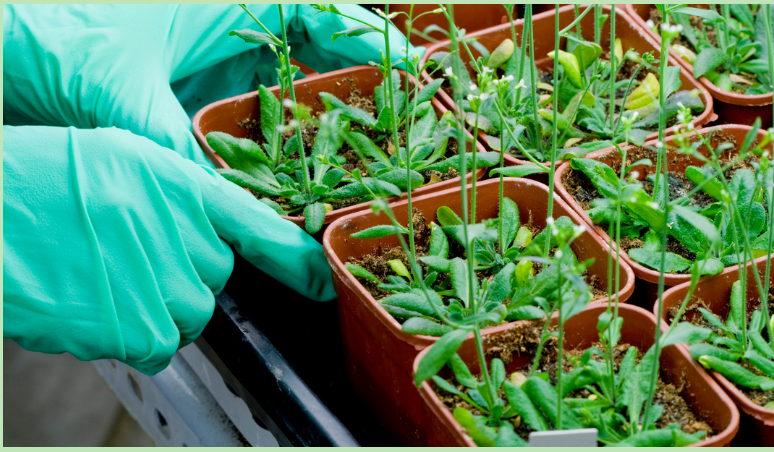

A single-cell *Arabidopsis* root atlas reveals developmental trajectories in wild type and cell identity mutants

Rachel Shahan[1,9], Che-Wei Hsu[2,3,9], Trevor M. Nolan[1,10], Benjamin J. Cole[4,10], Isaiah W. Taylor[1], Laura Greenstreet[5], Stephen Zhang[5], Anton Afanassiev[5], Anna Hendrika Cornelia Vlot[3,6], Geoffrey Schiebinger[5], Philip N. Benfey[1,7], and Uwe Ohler[2,3,8]
1 Department of Biology, Duke University, Durham, North Carolina 27708, USA
2 Department of Biology, Humboldt Universität zu Berlin, 10117 Berlin, Germany
3 The Berlin Institute for Medical Systems Biology, Max Delbrück Center for Molecular Medicine, 10115 Berlin, Germany
4 Department of Energy Joint Genome Institute, Walnut Creek, California 94598, USA
5 Department of Mathematics, University of British Columbia, Vancouver, V6T 1Z2 British Columbia, Canada
6 Technische Universität Berlin, Germany
7 Howard Hughes Medical Institute, Duke University, Durham, North Carolina 27708, USA
8 Department of Computer Science, Humboldt Universität zu Berlin, 10117 Berlin, Germany
9, 10 These authors contributed equally to this work

Spatiotemporal gene regulatory networks orchestrate organ development. Mapping global gene expression patterns across developmental time is essential to understand how these networks facilitate tissue patterning, cell identity acquisition, and terminal tissue differentiation. We are exploiting the simplifying properties of the *Arabidopsis* root, a stereotypically developing organ with indeterminate growth, immobile cells, and defined cell lineages, to construct an organ-scale gene expression atlas at single cell resolution. The atlas represents all major cell and tissue types over a finely resolved progression of developmental states. To build the atlas, we harmonized single cell mRNA sequencing data from over 110,000 cells. We applied stationary optimal transport, a variant of Waddington Optimal Transport (Schiebinger et al., 2019) for systems in equilibrium, to infer developmental trajectories and predict candidate transcriptional regulators. To test the utility of the atlas to interpret new datasets, we profiled mutants of two key transcriptional regulators at single cell resolution, *shortroot* and *scarecrow.* In addition to reflecting known tissue composition phenotypes for both mutants, the data suggest previously unappreciated trans-differentiation of cells from cortex to endodermis identity in the *scarecrow* mutant. Taken together, the atlas is a rich resource for unraveling the transcriptional programs that regulate root development in space and time.

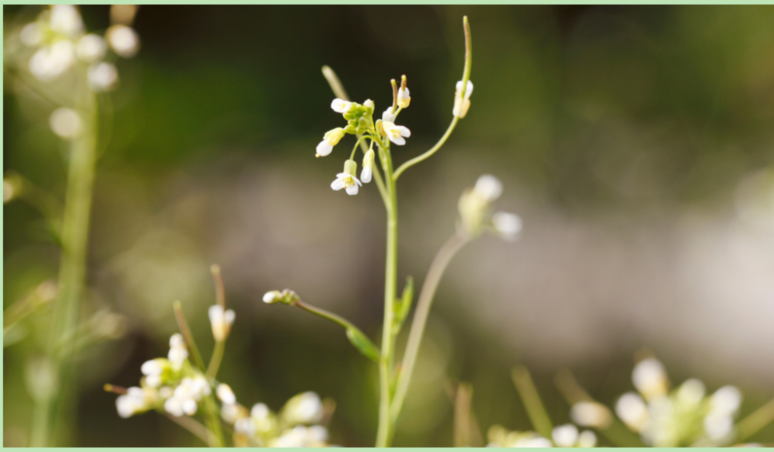

DR. ARGYRIS ZARDILIS

University of Cambridge-Sainsbury
Laboratory,
UK

Integrating molecular and mechanical signals in *Arabidopsis* early flower development

Argyris Zardilis, Yassin Refahi, Henrik Jönsson, Jan Traas

Genetic programming has always been recognised as the main basis of morphogenesis. This has led to many studies of genes that have various effects on morphogenetic processes. These results are, however, rarely integrated in space and time or linked to quantitative developmental outputs. Furthermore, purely genetic programming leaves a large explanation gap in morphogenetic processes since the gene-phenotype link is rarely direct and leaves no space for mechanical signals.
In this talk, we will first present recent work where we integrated patterns from 30 genes relevant in development into the same reference time series of *Arabidopsis thaliana* flowers [1]. This allowed us to get an integrated picture of their regulation in space and time and their link to growth. We will then present follow up work on linking these information with other recognised mechanical signals (stress, [2]) in biophysical models on realistic templates of the flower combined with experimental data on mechanical perturbations. The combination of chemical and mechanical signals will allow us to close the explanatory gap from cells to tissues for a more integrated picture of this developmental process.

1. Refahi, Yassin, et al. "A multiscale analysis of early flower development in Arabidopsis provides an integrated view of molecular regulation and growth control." *Developmental Cell* 56.4 (2021): 540-556.

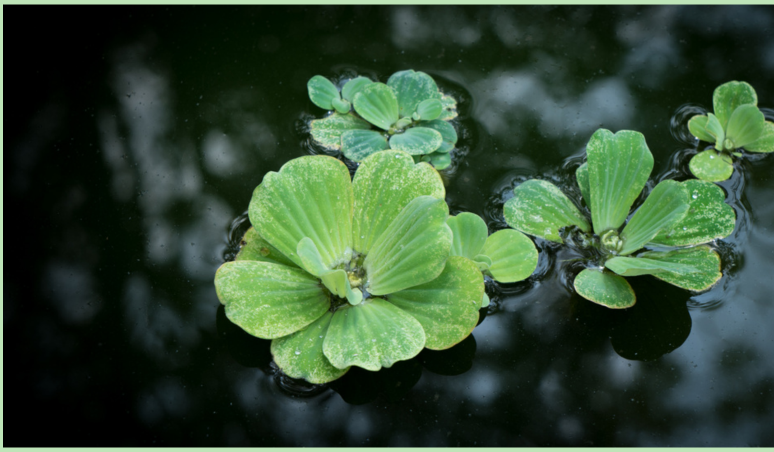

DR. BRADLEY ABRAMSON

Salk Institute for Biological Studies, USA

Single nuclei transcriptome of the Lesser Duckweed *Lemna minuta* reveals cell trajectories for an entire plant

Bradley W. Abramson, Mark Novotny, Nolan T. Hartwick, Kelly Colt, Brian D. Aevermann, Richard H. Scheuermann, Todd P. Michael

The ability to trace every cell in some model organisms has led to the fundamental understanding of development and cellular function. However, in plants the complexity of cell number, organ size and developmental times makes this a challenge even in the diminutive model plant *Arabidopsis thaliana.* Here we develop the Lesser Duckweed *Lemna minuta* as a model with a reduced body plan, small genome size and clonal growth pattern that enables simultaneous tracing of cells from the entire plant over the complete developmental cycle. We generated a chromosome-resolved genome for the 360 megabase genome and defined the growth trajectory of the entire plant with single nuclei RNA sequencing. The *L. minuta* gene complement represents a primarily non-redundant set with only the ancient *tau* whole genome duplication shared with all monocots, and paralog expansion as a result of tandem duplications related to phytoremediation. Thirteen distinct cell types representing meristem, the leaf-stem fusion called a frond, and root-like tissues were defined using gene orthology with single cell expression from model plants, gene ontology categories, and cell trajectory analysis. Dividing meristem cells give rise to two main branches of root-transition and mesophyll cells, which then give rise to terminally differentiated parenchyma, epidermal and root cells. Mesophyll tissues express high levels of elemental transport genes consistent with this tissue playing a role in *L. minuta* wastewater detoxification. The *L. minuta* genome and cell map provide a paradigm to decipher developmental genes and pathways for an entire plant.

DR. ZHONGPENG LI

Iowa State University,
USA

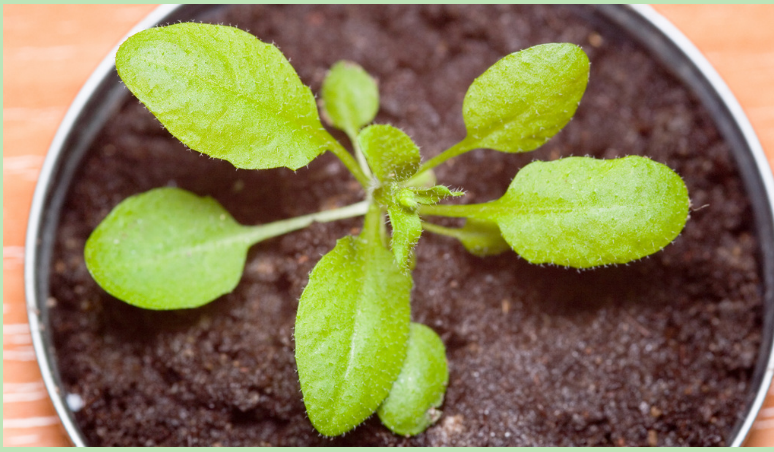

Regulation of plasmodesmata function at specific cell-cell interfaces in *Arabidopsis*

Zhongpeng Li and Kyaw Aung
Department of Genetics, Development, and Cell Biology, Iowa State University, Ames, IA 50014, USA

Precise exchange of information and resources among cells is essential for multicellular organisms. Despite the significance of intercellular communication, it is largely unknown how the communication between different cells is regulated. In plants, symplastic movement of molecules, including photosynthetic products, is governed by membrane-lined channels termed plasmodesmata (PD), which connect adjoining cells. The function of PD is controlled by homeostasis of a plant polysaccharide, callose. In addition, plasmodesmata-located proteins (PDLPs) play important roles in regulating the plasmodesmal function. Two independent methods, expression of fusion proteins driven by native promoters and GUS-reporter assay, demonstrate that PDLP5 and PDLP6 express in non-overlapping cell types in *Arabidopsis.* PDLP5 is mainly detected in leaf epidermis, whereas PDLP6 is expressed only in leaf vasculature, likely in phloem parenchyma cells. Overexpression of PDLP5 and PDLP6 lead to dwarfism and late flowering. Overexpression of PDLP5 results in starch overaccumulation in mesophyll cells in mature leaves. PDLP6 overexpressor, on the other hand, overaccumulates starch in both mesophyll cells and bundle sheath cells in mature leaves. The findings suggest that overexpression of PDLP5 and PDLP6 blocks the PD-dependent movement of sugars at different cell-cell interfaces. We also observed that overexpression of PDLP5 shows higher accumulation of callose at PD connecting epidermal cells and mesophyll cells in leaves, whereas overexpression of PDLP6 results in higher accumulation of callose only in vascular tissues. Together, our data indicate that PDLP5 and PDLP6 expression in and function at different cell-cell interfaces to regulate the plasmodemal function. Our work begins to reveal how communication among distinct cell types is regulated in plants. Further research should uncover the function of PDLPs and their partner proteins in regulating PD at the molecular level.

KARI MILLER

Washington University in St. Louis, USA

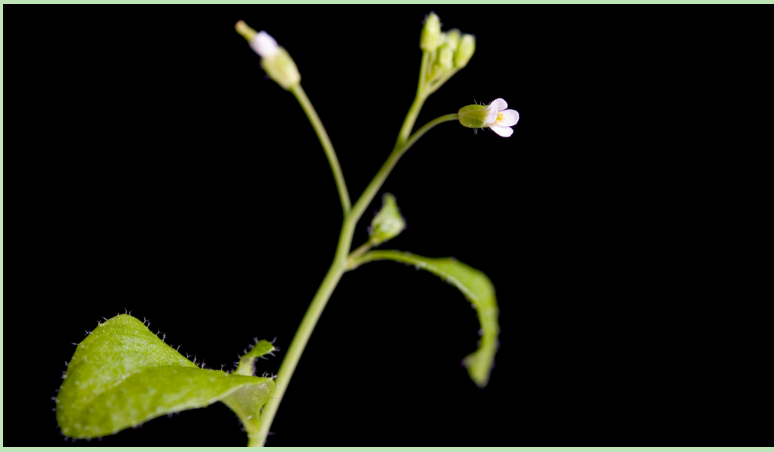

In vitro experiments and kinetic models of pollen hydration mechanics show that MSL8 is not a simple tension-gated osmoregulator

Kari Miller[1,2], Wanda Strychalski [3], Masoud Nickaeen[4], Anders Carlsson[2,5], Elizabeth S. Haswell[1,2]
1 Department of Biology, Washington University, St. Louis, Missouri 63130, USA
2 NSF Center for Engineering Mechanobiology
3 Department of Mathematics, Applied Mathematics, and Statistics, Case Western Reserve University, Cleveland, OH 44106, USA
4 University of Connecticut School of Medicine, Farmington, CT 06030, USA
5 Department of Physics, Washington University, St. Louis, Missouri 63130, USA

Pollen, a neighbor-less cell that contains the male gametes, undergoes multiple mechanical challenges during plant sexual reproduction, including desiccation and rehydration. It was previously showed that the pollen-specific mechanosensitive ion channel MscS-Like (MSL)8 is essential for pollen survival during hydration and proposed that it functions as a tension-gated osmoregulator. Here we test this hypothesis with a combination of mathematical modeling and laboratory experiments. Time-lapse imaging revealed that wild-type pollen grains swell and then stabilize in volume rapidly during hydration. *msl8* mutant pollen grains, however, continue to expand and eventually burst. We found that a mathematical model wherein MSL8 acts as a simple tension-gated osmoregulator does not replicate this behavior. A better fit was obtained from variations of the model wherein MSL8 inactivation is independent of its membrane tension gating threshold or MSL8 strengthens the cell wall without osmotic regulation. Experimental and computational testing of several perturbations, including hydration in an osmolyte-rich solution, hyper-desiccation of the grains, and MSL8-YFP overexpression, indicated that the Cell Wall Strengthening Model best simulated experimental responses. Finally, expression of a non conducting MSL8 variant did not complement the *msl8* overexpansion phenotype. These data indicate that, contrary to our hypothesis and to known MS ion channel function in single-cell systems, MSL8 does not act as a simple membrane tension-gated osmoregulator. Instead, they support a model wherein ion flux through MSL8 is required to alter pollen cell wall properties. These results demonstrate the utility of pollen as a cellular-scale model system and illustrate how mathematical models can correct intuitive hypotheses.

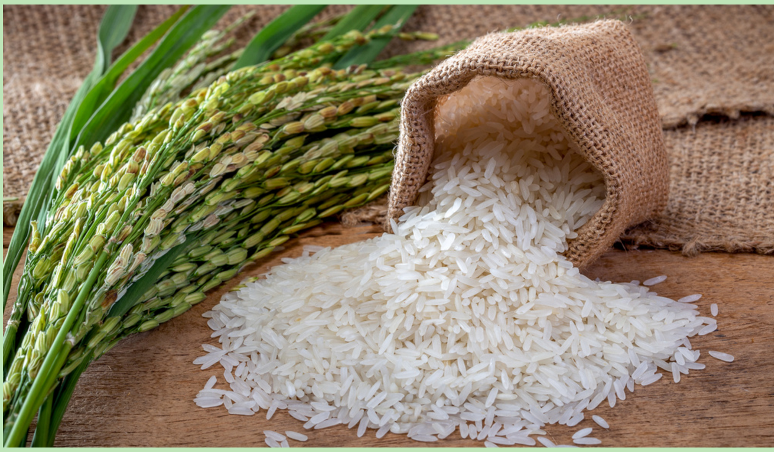

ALEX BOROWSKY

University of California, Riverside, USA

A field-grown rice translatome atlas

Mauricio A. Reynoso*, Alexander T. Borowsky*, Germain C. Pauluzzi*, Elaine Yeung, Jianhai Zhang, Elide Formentin, Joel Velasco, Sean Cabanlit, Christine Duvenjian, Matthew J. Prior, Garo Z. Akmakjian, Roger B. Deal, Neelima Sinha, Siobhan M. Brady, Thomas Girke, Julia Bailey-Serres
*Equal contributors

Crops uptake nutrients in the soil through roots, and genetic manipulation of root traits has the potential to improve nutrient uptake, increase tolerance to nutrient-poor soils, and increase yield. To better understand how rice roots develop and interact with the environment, we deployed Translating Ribosome Affinity Purification (TRAP) to mark translating ribosomes in specific cell populations in rice roots growing in a paddy field. After sequencing ribosome-associated mRNAs, we identified transcripts enriched in promoter-defined cell populations that represent different cell layers and domains of the rice root. mRNAs enriched in particular cell populations included genes responsible for specific metabolic pathways. Associating the enrichment of mRNAs for certain cell populations with the presence of DNA-cis motifs in corresponding promoter regions also reveals transcription factors that likely play roles in defining the identity of these cell populations. We also compare the cell population enriched genes to profiling we have conducted across different environments to identify stable cell type markers. This knowledge can provide insight for approaches to engineering rice that is tolerant to abiotic stresses.

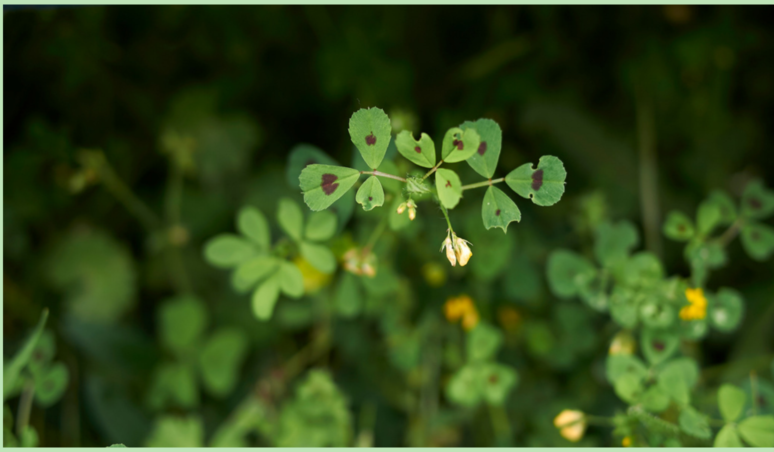

KAREN SERRANO

Lawrence Berkeley National Laboratory, USA

The Plant-Fungal Interface: Application of Spatially-Resolved, Single Cell Transcriptomics to the Arbuscular Mycorrhizal Symbiosis

Karen Serrano[1], Margot Bezrutczyk[2] , Danielle Goudeau[2] , Rex Malmstrom[2] , Ronan O'Malley[2] ,
Axel Visel[2] , Henrik Scheller[1], Benjamin J. Cole[2]
1 Joint BioEnergy Institute, Emeryville, California, USA
2 DOE Joint Genome Institute, Lawrence Berkeley National Lab, Berkeley, California, USA

The arbuscular mycorrhizal (AM) symbiosis is critical to land plant productivity. Studies of genome-scale reprogramming using bulk- and Laser Capture Microdissection-based transcriptome profiling methods demonstrated that dramatic shifts occur in both plant and fungal transcriptomes during AM symbiosis establishment. A major limitation to these studies is that they require pooling together cells of unique AM developmental stages, thus reducing the sensitivity of the analysis to detect stage-specific transcripts. The objective of this study is to apply the spatially-resolved, near-single-cell transcriptomics technique (Visium, 10X Genomics) to the analysis of the symbiosis between *Medicago truncatula* and *Rhizophagus irregularis.* Spatial and single-cell transcriptomic data from will be combined with AM-phenotypic results for both colonized and naive roots from wild-type plants and CRISPR-generated *gint1* mutants. MtGINT1 is an N-acetylglucosamine transferase that functions in the biosynthesis of major plasma membrane lipids in plants. The *gint1* mutant shows abnormal arbuscule development. Analysis of *gint1* compared to control plants will identify and characterize genes that are differentially expressed between 1) inoculated and control wild-type and *gint1* roots 2) colonized and non-colonized cells and 3) AM fungal structures. Candidate loci will be validated for their expressions and function in AM symbiosis. The outcome will generate greater understanding of both plant and fungal genes that control arbuscule development at an unprecedented scale.

ACKNOWLEDGEMENTS

SPEAKERS

AMIR H. AHKAMI (PACIFIC NORTHWEST NATIONAL LABORATORY, USA)
VIMAL KUMAR BALASUBRAMANIAN (PACIFIC NORTHWEST NATIONAL LABORATORY, USA)
MARGOT BEZRUTCZYK (LAWRENCE BERKELEY NATIONAL LABORATORY, USA)
FEDERICA BRANDIZZI (MICHIGAN STATE UNIVERSITY, USA)
MAITE COLINAS (MAX PLANCK INSTITUTE FOR CHEMICAL ECOLOGY, GERMANY)
KEVIN COX, JR (DONALD DANFORTH PLANT SCIENCE CENTER, USA)
CAMILLA FERRARI (VIB-GENT UNIVERSITY, BELGIUM)
NANCY GEORGE (EUROPEAN MOLECULAR BIOLOGY LAB - EBI, UK)
STEFANIA GIACOMELLO (SCILIFELAB - KTH, SWEDEN)
BRUNO GUILLOTIN (NEW YORK UNIVERSITY, USA)
YVON JAILLAIS (ENS LYON, FRANCE)
CONSTANCE LE GLOANEC (UNIVERSITY OF MONTREAL, CANADA)
MATHEW LEWSEY (LA TROBE UNIVERSITY, AUSTRALIA)
MARC LIBAULT (UNIVERSITY OF NEBRASKA, LINCOLN, USA)
HILDE NELISSEN (VIB-GENT UNIVERSITY, BELGIUM)
LACHEZAR NIKOLOV (UNIVERSITY OF CALIFORNIA, LOS ANGELES, USA)
GERGO PALFALVI (NATIONAL INSTITUTE FOR BASIC BIOLOGY, JAPAN)
IRENE PAPATHEODOROU (EUROPEAN MOLECULAR BIOLOGY LAB - EBI, UK)
MICHAEL TAYLOR (PACIFIC NORTHWEST NATIONAL LABORATORY, USA)
XIAOSA XU (COLD SPRING HARBOR LABORATORY, USA)

MODERATORS

CHRISTOPHER ANDERTON (PACIFIC NORTHWEST NATIONAL LABORATORY, USA)
ALEX BOROWSKY (UNIVERSITY OF CALIFORNIA, RIVERSIDE, USA)
BENJAMIN COLE (LAWRENCE BERKELEY NATIONAL LABORATORY, USA)
SHAO-SHAN CAROL HUANG (NEW YORK UNIVERSITY, USA)
KAISA KAJALA (UTRECHT UNIVERSITY, NETHERLANDS)
SAM LEIBOFF (OREGON STATE UNIVERSITY, USA)
MARC LIBAULT (UNIVERSITY OF NEBRASKA, LINCOLN, USA)
GERGO PALFALVI (NATIONAL INSTITUTE FOR BASIC BIOLOGY, JAPAN)
RACHEL SHAHAN (DUKE UNIVERSITY, USA)
SHOULING XU (CARNEGIE INSTITUTION FOR SCIENCE, USA)

ACKNOWLEDGEMENTS

ORGANIZERS

CHRISTOPHER ANDERTON (PACIFIC NORTHWEST NATIONAL LABORATORY, USA)
KEN BIRNBAUM (NEW YORK UNIVERSITY, USA)
JENNIFER BROPHY (STANFORD UNIVERSITY, USA)
BENJAMIN COLE (LAWRENCE BERKELEY NATIONAL LABORATORY, USA)
DIANE DICKEL (LAWRENCE BERKELEY NATIONAL LABORATORY, USA)
DAVID EHRHARDT (CARNEGIE INSTITUTION FOR SCIENCE, USA)
NOAH FAHLGREN (DONALD DANFORTH PLANT SCIENCE CENTER, USA)
MARGARET FRANK (CORNELL UNIVERSITY, USA)
ELIZABETH HASWELL (WASHINGTON UNIVERSITY IN ST. LOUIS, USA)
SHAO-SHAN CAROL HUANG (NEW YORK UNIVERSITY, USA)
ELENA LAZARUS (CARNEGIE INSTITUTION FOR SCIENCE, USA)
SAMUEL LEIBOFF (OREGON STATE UNIVERSITY, USA)
MARC LIBAULT (UNIVERSITY OF NEBRASKA, LINCOLN, USA)
MARISA OTEGUI (UNIVERSITY OF WISCONSIN, USA)
NICHOLAS PROVART (UNIVERSITY OF TORONTO, CANADA)
SUE RHEE (CARNEGIE INSTITUTION FOR SCIENCE, USA)
SELENA RICE (CARNEGIE INSTITUTION FOR SCIENCE, USA)
R. GLEN UHRIG (UNIVERSITY OF ALBERTA, CANADA)

CARNEGIE SCIENCE

THANK YOU FOR ATTENDING!

NSF

STOCK PHOTOS FROM CANAVA AND EARTH.COM
